# Effect of climate on strategies of nest and body temperature regulation in paper wasps, *Polistes biglumis* and *Polistes gallicus*

**DOI:** 10.1038/s41598-022-07279-0

**Published:** 2022-03-01

**Authors:** Anton Stabentheiner, Julia Magdalena Nagy, Helmut Kovac, Helmut Käfer, Iacopo Petrocelli, Stefano Turillazzi

**Affiliations:** 1 Institute of Biology, University of Graz, Universitätsplatz 2, 8010 Graz, Austria; 2 Department of Neuroscience and Developmental Biology, University of Vienna, Djerassiplatz 1, 1030 Wien, Austria; 3 Dipartimento di Biologia, Università di Firenze, Via Madonna del Piano 6, 50019 Sesto Fiorentino, Italy

**Keywords:** Ecophysiology, Animal behaviour

## Abstract

*Polistes* paper wasps are a widespread taxon inhabiting various climates. They build nests in the open without a protective outer layer, which makes them vulnerable to changing temperatures. To better understand the options they have to react to environmental variation and climate change, we here compare the thermoregulatory behavior of *Polistes biglumis* from cool Alpine climate with *Polistes gallicus* from warm Mediterranean climate. Behavioral plasticity helps both of them to withstand environmental variation. *P. biglumis* builds the nests oriented toward east-south-east to gain solar heat of the morning sun. This increases the brood temperature considerably above the ambience, which speeds up brood development. *P. gallicus*, by contrast, mostly avoids nesting sites with direct insolation, which protects their brood from heat stress on hot days. To keep the brood temperature below 40–42 °C on warm days, the adults of the two species show differential use of their common cooling behaviors. While *P. biglumis* prefers fanning of cool ambient air onto the nest heated by the sun and additionally cools with water drops, *P. gallicus* prefers cooling with water drops because fanning of warm ambient air onto a warm nest would not cool it, and restricts fanning to nests heated by the sun.

## Introduction

Polistine wasps are distributed across quite different climates all over the world^1–10^. Temperature as a main abiotic factor influences many physiological and biochemical processes of animals^11^. As a consequence, it has a major impact on the distribution of insects^12,13^. In a variable environment, appropriate strategies of thermoregulation are essential to achieve optimal development conditions for the brood. In social wasp and bee societies it is the adults which have to take care of optimal brood conditions. The thermoregulatory strategies of brood care can be based on behavioral or metabolic measures^14^. If adults of social insects activate their flight muscles the brood can benefit from the emitted heat (e.g.^15^). In *Polistes* paper wasps, however, any active (metabolic) heating effort of the adults would mean much wasted energy because the heat is immediately lost to the surrounding air^14,16–19^. Therefore, they have to regulate the nest temperature by behavioral means, which includes nest site choice as an initial, ‘forward-looking’ strategy^20–24^. By proper nest site choice in sheltered places *Polistes dominula* from cool temperate climate, for example, is able to achieve a nest climate not much different from that of open-nesting *Polistes gallicus* from the warmer Mediterranean climate^25^. In addition to nest site choice, ‘immediate’ behavioral means like cooling by fanning and distribution of water droplets on the nest for evaporative cooling allow control of the nest temperature^16,20^.

In view of climate change, changes in mean annual temperature will affect ecosystems and thus the dispersion and survival of insects^13,25–32^. Even minimal changes could have a crucial effect on the development of the individuals^4,12,20,33–35^. Brood development is especially dependent on environmental conditions in open-nesting species like *Polistes gallicus* and *Polistes biglumis*. Polistine wasps inhabiting differing climates are expected to show differing reaction norms and sensitivities to temperature^36,37^. Adults of *Polistes biglumis* inhabiting Alpine climate, for example, have a considerably lower resting energy turnover and sensitivity to temperature changes than *Polistes gallicus* inhabiting Mediterranean climate^36^. We hypothesize that the differences in climate which these two open-nesting paper wasps experience also influence their behavioral strategies of nest thermoregulation. Behavioral plasticity may promote both immediate (short-term) reaction to environmental variation and long-term resistance to climatic changes due to global change, without the need of immediate adaptation of physiological traits. To shed light on the thermoregulatory ability of these *Polistes* species, therefore, we here compare the effect of environmental difference and variation in temperature and radiation on these wasps’ body and brood temperature, on their behavioral measures of brood temperature control, and on their choice of nesting sites.

## Materials and methods

### Animals

We chose two closely related species which differ in their distribution and climatic preference. *P. biglumis* is found in montane areas in the Alps and the Apennines, whereas *P. gallicus* is located in Mediterranean climate regions^2,34,38,39^. Both are primitively eusocial wasps which do not build closed nests. In both of them brood developoment depends strongly on the thermal environment.

### Research locations

**Figure 1:**
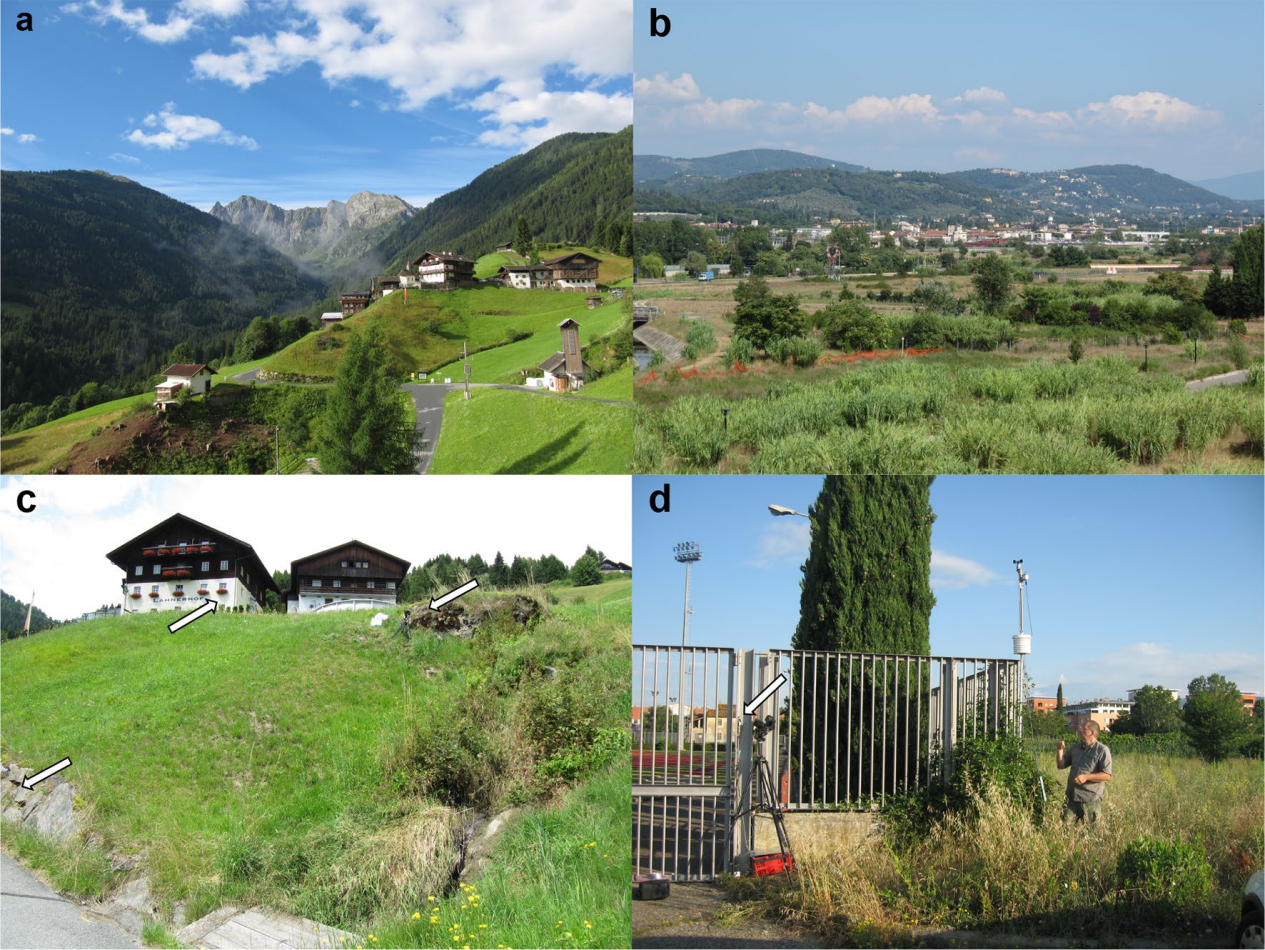
Research locations (top) with typical nesting sites (bottom, see arrows): (**a,c**) Obergail; (**b**,**d**) Sesto Fiorentino.

The main locations for observing *P. biglumis* were located in Austria, for the greater part in Obergail (Long. (°E)/Lat. (°N) = 12.79647/46.68904) (Lesachtal, Carinthia), and in Teichalm (15.443466/47.354827) and Krakauebene (13.95622/47.18892) (Styria). *P. gallicus* was investigated in Sesto Fiorentino in Italy (11.20688/43.81750) (Tuscany). Obergail (Fig. 1a,c), Teichalm and Krakauebene are mountainous regions ~ 1000–1450 m above sea level with a typical Alpine climate, with sometimes relatively warm days and often cold nights during midsummer. Sesto Fiorentino (Fig. 1b,d), only ~ 55 m above sea level, shows a Mediterranean climate with very high temperatures during midsummer. Temperatures do not decrease much during the night and often rise above 40 °C during the day. Mean annual temperatures 1981–2010 of nearest weather stations for Obergail (1160 m ASL), Teichalm (1150 m ASL) and Krakauebene (1313 m ASL) were: 6.9 °C in Kornat (990 m ASL), 4.3 °C on Schöckl mountain (1443 m ASL), and 5.2 °C in Tamsweg (1025 m ASL), respectively. The average of these mean annual temperatures (mean height = 1153 m ASL) amounts to ~ 5.4 °C^40^. Mean annual temperature 1981–2010 in Firenze (50 m ASL), the nearest weather station to Sesto Fiorentino (55 m ASL), was 15.2 °C^41^.

### Measurement of body and nest temperature

Measurements of body and nest temperatures were conducted in the summer seasons, in 7 inhabited nests of *P. biglumis* in Austria, in Obergail in the alpine valley of Lesachtal in 2010, 2017 and 2018 (12 days), and 2 empty nests in Teichalm (Styria) in 2019 (2 days). Measurements in *P. gallicus* were conducted in 10 inhabited and 6 empty nests in Sesto Fiorentino (near Firenze, Italy) in 2015, 2017 and 2018 (11 days; see Supplementary Table S1).

The surface temperature of the wasps (head, thorax, abdomen), of the brood (eggs, larvae and (prae)pupae) and of the whole nests was measured by infrared thermography, without touching and impairing the wasps or the nests, mostly with a FLIR T650sc camera (resolution 640 × 480 pixels, sensitivity < 20 mK), and sometimes with a FLIR i60 (resolution 180 × 180 pixels, sensitivity < 100 mK) (FLIR Systems Inc., Danderyd, Sweden). The measurement accuracy was ~ 0.7 °C, assuming a wasp cuticle infrared emissivity of 0.97^42–44^, a nest material emissivity of 0.94, and a stone or concrete emissivity of 0.93 for the nest background. Camera accuracy was controlled with a self-manufactured Peltier-driven reference source of known temperature and emissivity (accuracy < 0.3 °C^45^). Infrared data were stored digitally on internal memory cards and evaluated later in the laboratory.

For body and nest temperature measurements, thermograms were taken during daytime and overnight, at a rate of 1/min or every 10 s with the T650sc, or at random intervals with the i60. For body and nest temperature measurements during fanning events thermograms were recorded at a rate of 30 Hz with the T650sc. Evaluation of the surface temperatures of head (T_head_), thorax (T_thorax_), and abdomen (T_abdomen_) was done with FLIR ThermaCam Researcher Pro 2.10 (FLIR Systems Inc., Wilsonville, USA), controlled by a self-written Excel (Microsoft Corporation, Redmond, USA) VBA macro which also extracted the microclimatic data from the logger files at the time of thermographic measurement. Size and representative placement of software evaluation tools are shown in supplementary Fig. S1c,d.

Microclimate data was collected with data-loggers (ALMEMO 2690, Ahlborn GmbH, Holzkirchen, Germany). The actual ambient temperature beside the nests (within ~ 0.5–1 cm) was measured with NiCr/Ni thermocouples, and global radiation with Ahlborn ‘FLA613-GS mini spezial’ sensors (Ahlborn). Temperature measurements were corrected for the heating effect of solar radiation on the thermocouples. Relative humidity and ambient temperature in shade was measured within several meters of the nests with FHA 646-11 sensors (Ahlborn).

### Nest orientation

Nest orientation was determined in three locations in Austria (Obergail, Teichalm and Krakauebene) and one region in Italy (in and around Sesto Fiorentino). Horizontal orientation of nests was determined with a compass according to the axis of the central cells^34^. Vertical nest orientation was determined with a goniometer, using the nest pedicel as a reference.

### Statistics

Simple regression analysis and graphing was done with Origin 2017 (OriginLab). Multiple regression statistics (modelling the dependence of nest and wasp body temperatures on environmental factors) and multifactor ANOVA (to test for differences of nest and body temperature control between species after compensation of environmental variables) was done with Statgraphics Centurion 18 software (Statgraphics Technologies). Calculation of mean and median angles of nest orientation was done according to Batschelet^46^.

## Results

**Figure 2:**
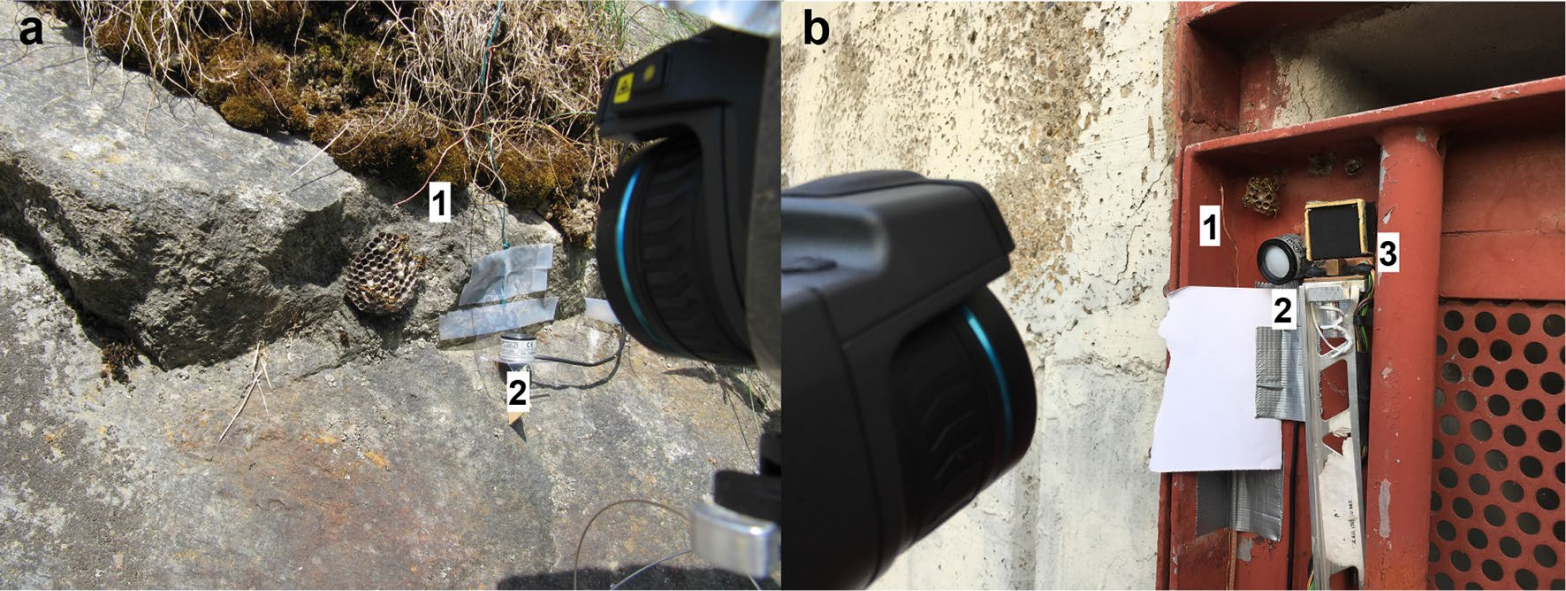
Examples of nests and fieldwork set-up in Obergail (**a**) and Sesto Fiorentino (**b**). 1 = thermocouple wire; 2 = global radiation sensor, 3 = Peltier-element IR reference source.

Both in *Polistes biglumis* and *P. gallicus* in most of the inhabited nests all types of brood were present: eggs, larvae and pupae (Table S1), with the exception of one foundress nest of *P. biglumis* with only one egg. The size of thermographed nests was quite variable in both species, the number of cells ranging from 18 to 99 in *P. biglumis* (mean: 61.6 cells), and from 19 to 381 in *P. gallicus* (mean: 101.7 cells) (Table S1). The mean number of wasps on the thermographed nests was higher in *P. gallicus* (12.6 wasps) than in *P. biglumis* (7.1 wasps). All nests of *Polistes biglumis* we observed in this study were built on stone substrate or walls (Figs. 1c, 2a). Only recently we found one nest built on a pile of wood. The choice of the nest substrate was more diverse in *P. gallicus* (Figs. 1d, 2b). They chose stone, concrete, walls, window grilles, and metal of fences or doorframes.

### Daily nest temperature course

#### Polistes biglumis

**Figure 3:**
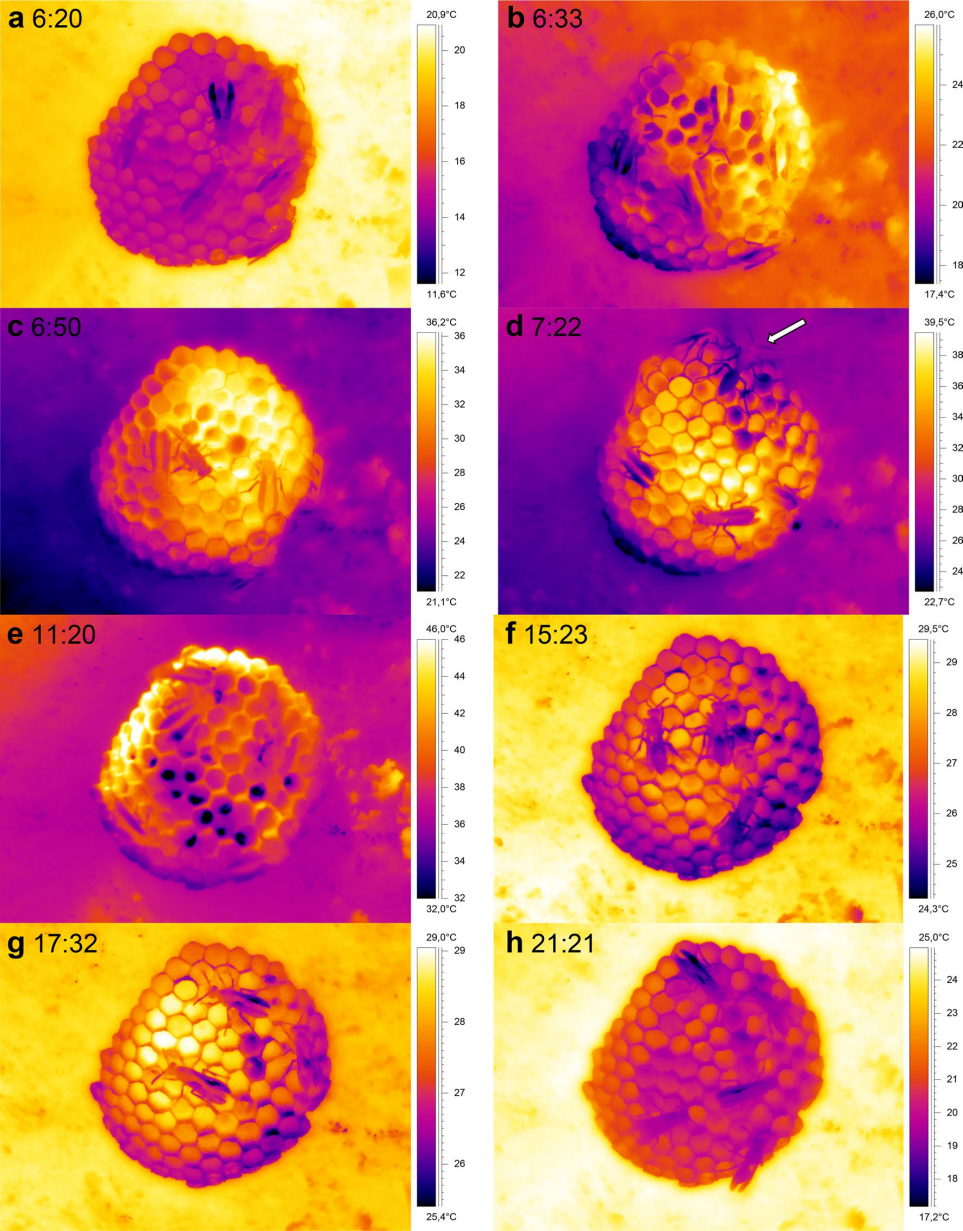
Thermograms of a *P. biglumis* nest during a whole day (19.07.2017). (**a**) Before sunrise at 6:20; (**b**) during sunrise (06:33); (**c**) nest temperature increasing fast in sunshine; (**d**) with a fanner for convective nest cooling (arrow; see also Fig. S4); (**e**) with water drops for evaporative cooling when sunshine increased part of the nest to temperatures > 45 °C; (**f,g**) after sunset (nest now in shade) in the afternoon; (**h**) at dusk with wasps sitting motionless on the nest. Time = CEST = UTC + 2 h.

Figure 3 shows a sequence of thermograms of a *P. biglumis* nest taken from dawn to dusk. Before sunrise the temperatures of the nest and of the wasps on it were quite low (mean ~ 15 °C) and uniform (~ 12 to 17.5 °C; Fig. 3a). The temperature of the stone substrate where the nest was built on was considerably higher (~ 20 °C). After sunrise (Fig. 3b,c) the nest temperature began to rise quickly. It only needed 13 min of sunshine (radiation) to heat the nest from ~ 17 to ~ 25 °C. Within one hour, temperature differences of almost 20 °C were measured within the nest. At 6:50, when the highest temperature on the nest was already at 36.2 °C, fast movements of the adults with inspections of the cells were observed (Fig. 3c). Soon afterwards the increasing temperature induced the wasps to start fanning (arrow in Fig. 3d). The wasps also began to gather water and spread it on and inside cells to cool the nest by evaporation (Fig. 3d,e). Towards late morning, some parts of the nest reached temperatures as high as 46 °C (Fig. 3e)! As soon as the nest was shaded by the substrate (~ 13:00) the nest temperature decreased according to the decrease in ambient temperature (Fig. 3f,g), reaching ~ 21 °C on average after dusk (Fig. 3h). At that time the substrate temperature (~ 25 °C) was still about 4 °C higher than the nest temperature.

**Figure 4:**
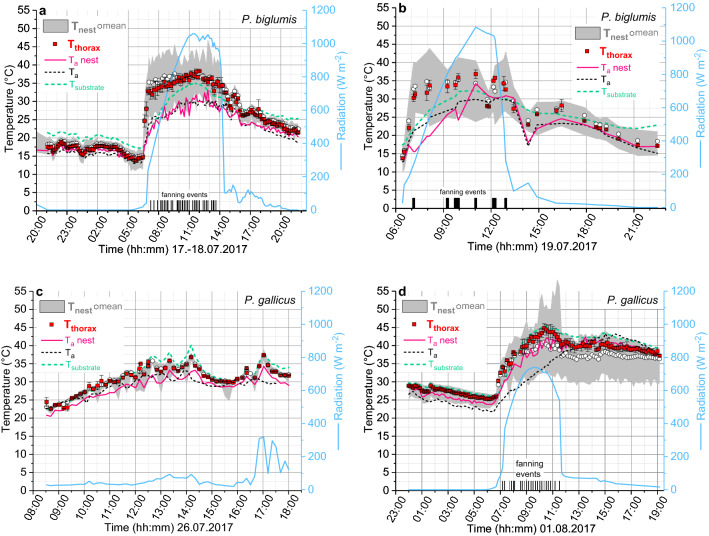
Examples of daily temperature changes of nests and wasps of *P. biglumis* (**a,b**) and *P. gallicus* (**c,d**). T_thorax_ = mean thorax surface temperature of up to five adult individuals per time of measurement; gray ribbon: total range of nest temperatures (T_max_:T_min_) with mean; T_substrate_ = temperature beside the nest (see Fig. S1c,d); T_a_nest = ambient air temperature directly at the nest. T_a_ = ambient air temperature in shade 1–3 m away from nest; Radiation = global radiation hitting the nest; black bars = fanning events at the time of thermographic measurements: actually, many more fanning events were observed. (**c**) Fanning was never observed! See also Fig. S2 for another example of a *P. gallicus* nest in shade. Time = CEST = UTC + 2 h.

The nest and body temperatures of a complete 24 h cycle of a different nest are shown in Fig. 4a. At night the nest temperature and the wasps’ thorax temperature decreased slowly according to the decrease of the air temperature. The substrate temperature was always higher than the mean nest temperature, which surely helped to keep the nest temperature higher than the temperature of the surrounding air (T_a_nest). Variation of within-nest temperature (max–min) was low at night. As soon as solar radiation increased in early morning, the nest temperature and the body temperature of the wasps on it increased rapidly, and the variation of nest temperature (max–min) increased (see also Fig. 3b). Though the maximum nest temperature reached values as high as 46.9 °C, cooling measures of the wasps (fanning and spreading of water drops, see below) kept the mean nest temperature always below 38.5 °C. Cooling of the nest after sunset (at the nest) was much slower than the increase in the morning, following the decrease of ambient and substrate temperature (Fig. 4a,b).

#### Polistes gallicus

Most *P. gallicus* nests were built in locations with no or only little direct sunshine (Figs. 2b, 4c, Fig. S2). In their habitats temperatures in midsummer are often already quite high in the morning, and may increase to values higher than 40 °C during the day (Fig. 4d). Mean temperatures of the nest and of the imagines on it were usually higher than the air temperature close to the nest (T_a_nest). In most nests variation of within-nest temperature (max–min) remained small throughout the day. On hot days (T_a_nest > 40 °C), however, maximum temperatures of empty cells in the nest margin sometimes reached values as high as 49.9 °C even in shade. Body temperature of the adults was mostly similar to the mean nest temperature (Fig. 4c, Fig. S2). At night, the nest temperature decreased according to the decrease of T_a_nest, similar to *P. biglumis* but at a higher level (Fig. 4d).

**Figure 5:**
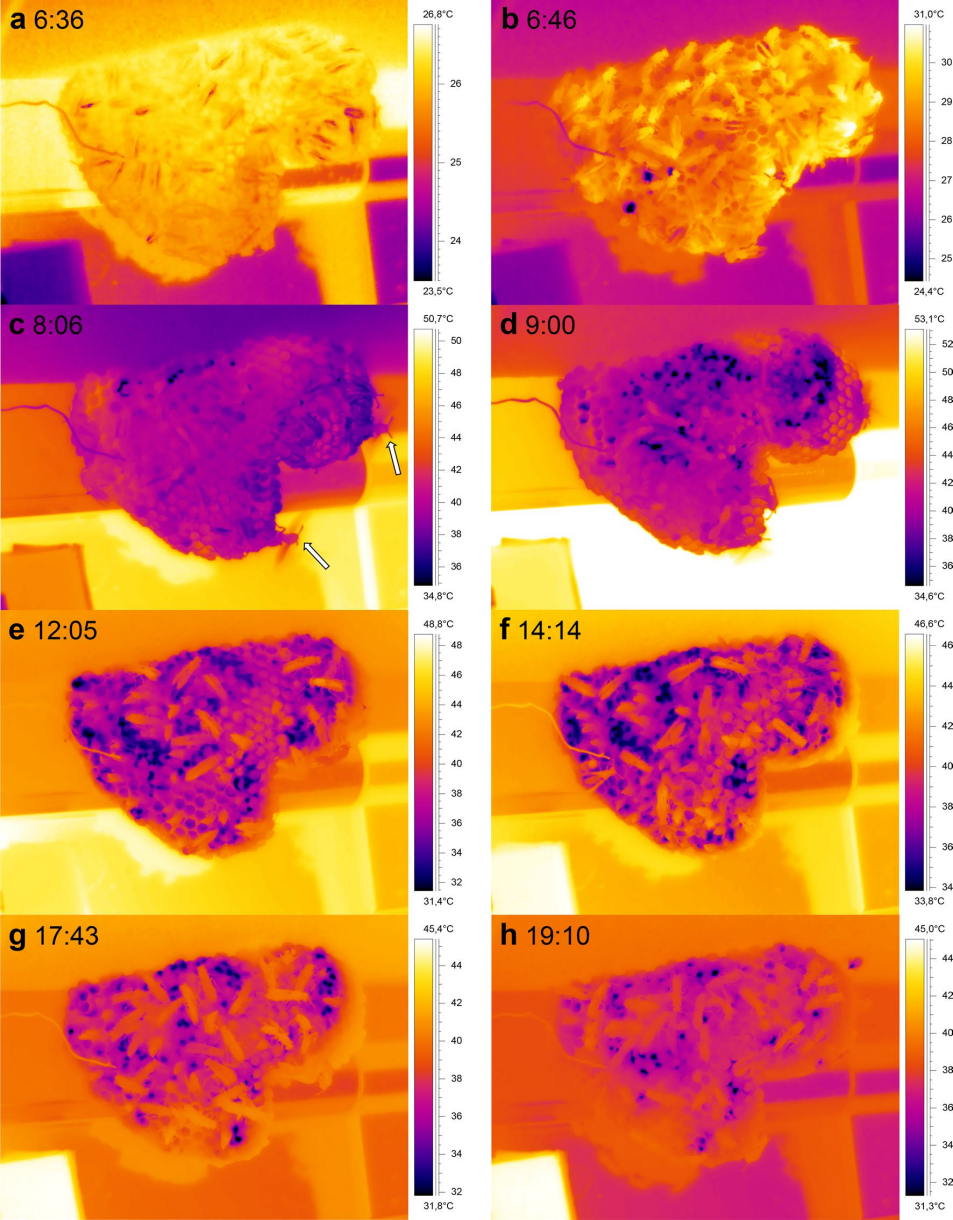
Thermograms of a large *P. gallicus* nest during a whole day (01.08.2017). Thermograms are rotated 90° clockwise (the upper part is on the right). (**a**) Before sunrise (6:36); (**b**) during sunrise (06:46) with the first water drops visible (dark spots); (**c**) with two fanners for convective nest cooling (arrows, see also Fig. 4d); (**d**) with more cooling drops; (**e**) after sunset at the nest site (nest now in shade); (**f–h**) after sunset in the afternoon and evening. Time = CEST = UTC + 2 h. For temperature evaluation see Fig. 4d.

The situation was different in one large nest which had been built in a location exposed to the morning sun (Figs. 4d, 5). On a hot day when T_a_nest increased to values higher than 42 °C, the body temperature of the adults increased to values up to 5 °C higher than the mean nest temperature. Nevertheless, though the combined effects of high air temperature and intense insolation increased part of the nest to a temperature of ~ 58 °C (Fig. 4d), mean nest temperature was kept below 41 °C. This was accomplished by cooling with many water droplets in the cells (dark spots in Fig. 5), and by the occurrence of fanning during the period when the sun was shining on the nest (Fig. 4d; see arrows in Fig. 5c). Fanning, however, was quite rare in all the other observed nests, even during the hottest time of the day! Water droplets were carried onto this nest until evening (Fig. 5h), as at that time the nest temperature was still at about 35–38 °C.

### Body and nest temperatures

**Figure 6:**
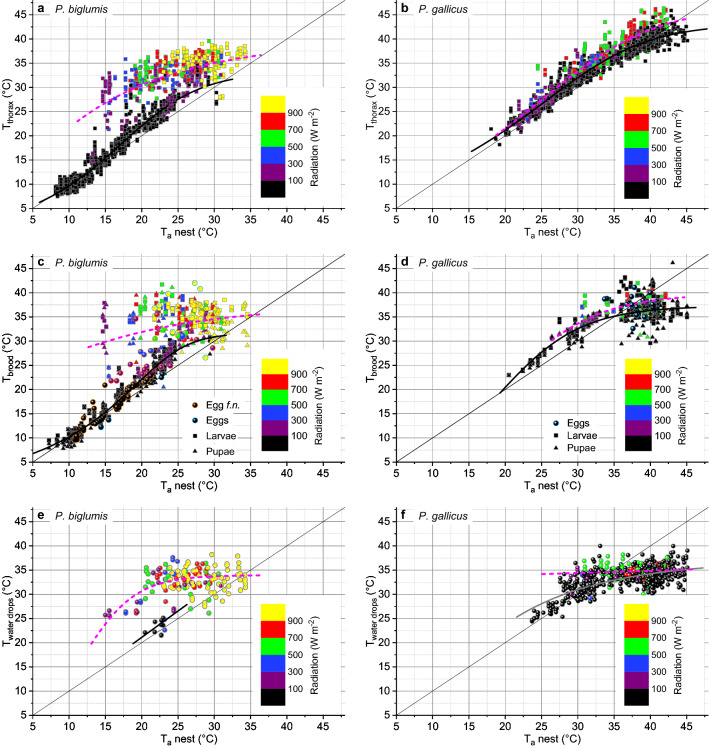
Surface temperature of the thorax of adult wasps, of different stages of brood and of water drops of *P. biglumis* (left) and *P. gallicus* (right), in dependence on ambient air temperature close to the nest (T_a_nest) and global radiation (color scale). Egg *f.n.* = single egg on a foundress nest; diagonal lines = isolines. Regressions were calculated for shaded conditions (Radiation = 0–100 W/m^2^; black or gray solid lines) and sunshine (Radiation > 100 W/m^2^; pink broken lines); P <<< 0.0001 for all except for linear regression in (**f**) (P = 0.021, pink line). For regression functions and detailed statistics see Table S2. Part of T_thorax_ values in *P. gallicus* included from Kovac et al.^25^.

**Figure 7:**
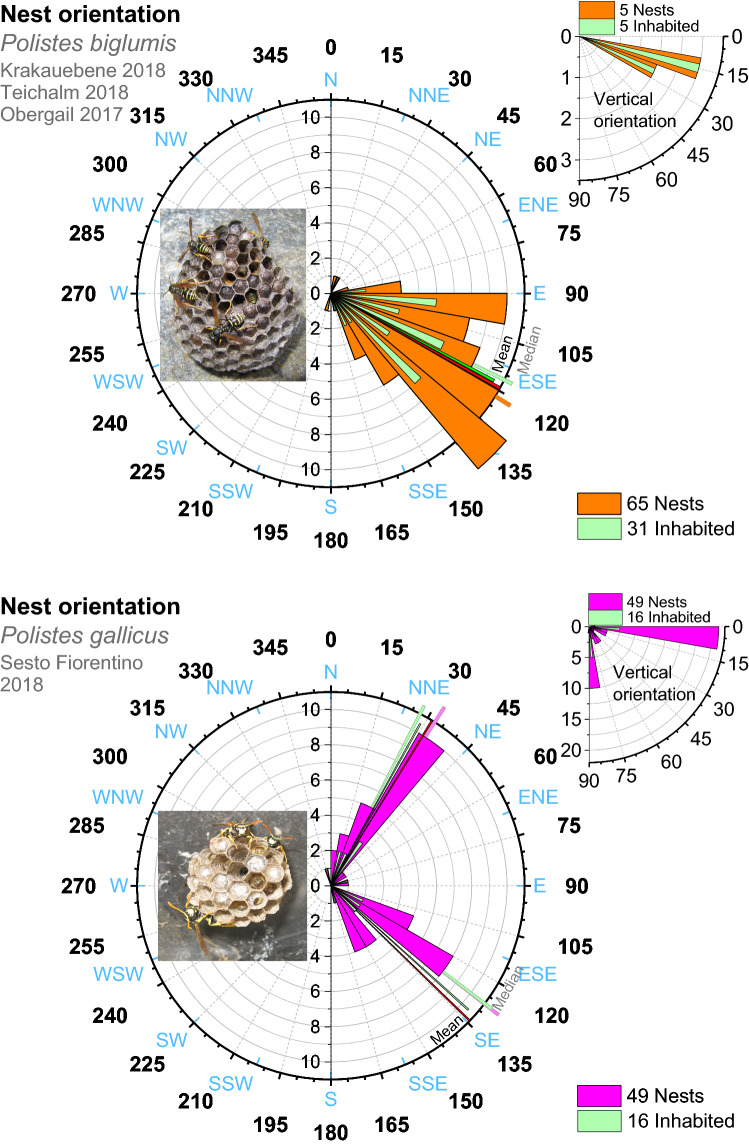
Horizontal and vertical nest orientation of *Polistes biglumis* in Alpine climate, and of *Polistes gallicus *in Mediterranean climate. Mean values and Medians (thin bars) calculated according to the rules of circular statistics^46^. Note the bifurcated distribution of nest orientation in *P. gallicus*.

Figure 6 shows a comparison of the dependence of body and nest temperatures on ambient air temperature and insolation between the two species. In the lower ranges of air temperature, usually at night, body temperature followed T_a_nest closely in both species. The exposition of the *P. biglumis* nests to the morning sun at ESE (Fig. 7) increased the wasp body temperature to values of often more than 15 °C higher than the surrounding air. However, body temperatures remained always below 40 °C (Fig. 6a). In *P. gallicus*, by contrast, the body temperature of the wasps increased considerably above 40 °C, to maximum values of about 46 °C, especially (but not exclusively) during intense insolation in the nest exposed to the morning sun (Fig. 6b).

Multiple linear model regression proved a significant influence of both ambient temperature close to the nest (T_a_nest) and global radiation on the temperature of thorax, head and abdomen in both *P. biglumis* and *P. gallicus* (P << 0.0001, ANOVA; Table S3). The same relationship was found for the temperature of the larvae and pupae (Table S3). For the mean nest temperature, including the brood, empty cells and the wasps on it (see “Poly” in Fig. S1c), the relation can be described by the multiple linear model equation T_nest_(mean) = 2.38935 + 1.0085 × T_a_nest + 0.00696722 × Radiation in *P. biglumis*, and T_nest_(mean) = 6.58421 + 0.821208 × T_a_nest + 0.00265919 × Radiation in *P. gallicus*, with R^2^ (adj. for df, %) = 82.3 and 83.5, respectively (P <<< 0.0001, ANOVA; for details and more model regressions see Table S3).

An extended model including the substrate temperature (where the nests were built on; see Fig. S1c,d) explained an even greater part of the total variation, with R^2^ (%) = 85.1 in *P. biglumis* and 93.7 in *P. gallicus*. The model equations were: T_nest_(mean) = − 2.14936 + 0.510637 × T_a_nest + 0.00475538 × Radiation + 0.611022 × T_substrate_ in *P. biglumis*, and T_nest_(mean) = 8.01929 + 0.0986386 × T_a_nest + 0.00318776 × Radiation + 0.620503 × T_substrate_ in *P. gallicus* (see Table S4 for more details).

A multifactor ANOVA comparison between the two species, compensating for the identified main environmental effects on nest and body temperature (T_a_nest, Radiation, T_substrate_) uncovered interesting similarities between the two species (Table S5), despite the differences in climate. Mean compensated nest temperature (T_nest_(mean)), and compensated temperatures of empty cells and cells containing eggs or larvae did not differ between species (‘Mean T’ in Table S5). In both *P. biglumis* and *P. gallicus* the mean temperature of empty nests, being without the thermal control by the adults, increased in parallel with ambient temperature (Fig. S3a,b). Maximum temperatures of empty nests reached quite high values in sunshine, even in the cool alpine climate experienced by *P. biglumis* (> 45 °C; Fig. S3a).

### Cooling mechanisms and nest orientation

In contrast to the adult wasps’ body temperature, brood temperature in both species usually did not exceed 42.5 °C (Fig. 6c,d). However, according to the differences in climate, strategies of brood temperature control were partially different between the two species. In *P. biglumis*, living in cool climate, the adults were able to increase the brood temperature considerably above the ambient level on sunny days (Fig. 6c) by building their nests exposed to the morning sun, facing ESE on average (Fig. 7). Nevertheless, they started cooling measures soon after sunrise to prevent overheating of the brood. First, they started heavy fanning to cool the nest by convection (Fig. 4a,b; see below). In addition, they flew out to bring water drops to the nest (Fig. 3d,e). The temperature of the water drops remained below 38 °C even in bright sunshine (Fig. 6e).

In most nests of *P. gallicus* the foundresses had built their nests preferably in locations with little direct sunshine on the nest (Fig. 4c, Fig. S2). Nest orientation was more variable, showing double-peaked distributions both in the horizontal and in the vertical direction (Fig. 7). Evaporative cooling with water droplets was the predominant acute measure of thermoregulation. Despite ambient temperatures up to 45 °C, droplet temperature nearly always remained below 38 °C (Fig. 6f), which mostly kept the brood temperature below 42.5 °C (Fig. 6d).

**Figure 8:**
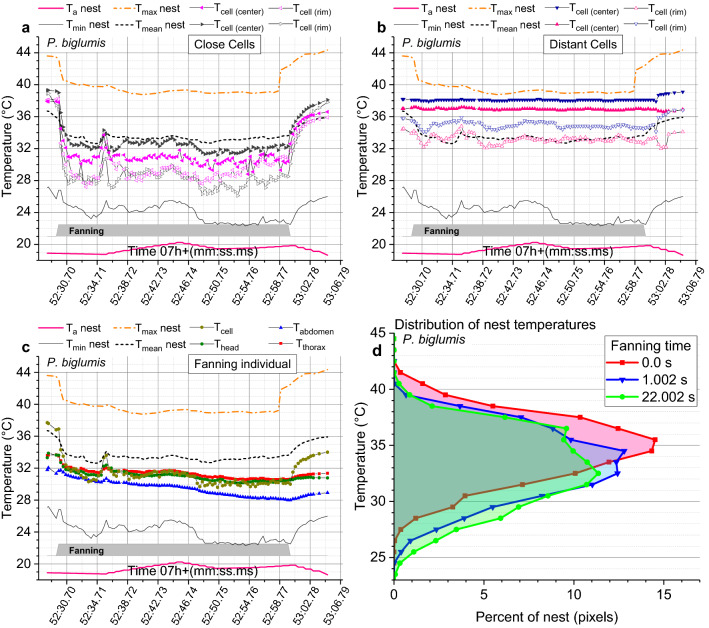
Cooling effect of fanning in a *Polistes biglumis *nest. Temperatures measured on cell rims and centers of cells close to and distant of the fanner, and of the body of the fanner. Also shown are maximum, mean and minimum nest temperature, air temperature close to the nest (T_a_nest), and in (**d**) the change of the total distribution of nest temperatures during different times of fanning (percent of nest at a certain temperature estimated from number of pixels in “Poly” in Fig. S2c). Time = CEST = UTC + 2 h. Gray bars = duration of fanning. For more samples see Figs. S5 and S6.

On warm sunny days, fanning was observed to be frequent in *P. biglumis*. It was rare in *P. gallicus*, occurring only when the nest was exposed to the sun (Fig. 4d). Therefore, its effect on nest cooling was investigated in *P. biglumis* in detail. Wasps engaged in fanning were often patrolling hectically across the nest, sometimes flying a small loop around the nest and again patrolling across the nest after landing (Fig. S4f, see Video S1). They frequently were putting their head inside cells for short periods, probably for temperature measurement with their antennal thermosensors (topmost wasp in Fig. S4b). Soon after start of fanning cell temperatures decreased around their position (Figs. 3d, 8, Figs. S4d, S5, S6).

In the example shown in Fig. 8 the temperature of cells close to the fanner experienced a sharp temperature drop within a second after start of fanning. The temperature decrease was higher at the cell rims (up to ~ 7–13 °C) than inside of the cells (up to ~ 6–9 °C) (Fig. 8a), and mostly much smaller in distant cells on the opposite part of the nest (Fig. 8b). Fanning also decreased the body temperature of the fanners by 1–2 °C on average, despite the activation of their flight muscles (Fig. 8c). After stop or interruption of fanning the cooling effect vanished quickly, and temperatures returned to the initial values within seconds (Fig. 8, Figs. S5, S6). Fanning shifted the temperatures of the whole nest by several degrees to lower values (Fig. 8d, Figs. S5, S6).

In an evaluation of 9 fanning events the mean cooling effect amounted to ~ − 2.4 °C and − 1.9 °C for maximum and mean nest temperature, respectively (Table 1). Rims and centers of cells close to the fanner decreased by − 5.7 °C and − 4.4 °C, whereas in cells distant to the fanner (on the opposite end of the nest) temperatures decreased by only − 0.7 °C and − 0.4 °C, respectively. It has to be kept in mind, however, that quite often more than one wasp was fanning at a time (see Video S1). The cooling effect on the nest may therefore often be spacially more extensive than shown in Figs. 3d, S4d and 8, and larger than reported in Table 1.

**Table 1:** Mean temperatures (± SD) during fanning events in *Polistes biglumis* nests exposed to the sun.

		Temperatures (°C)			
		Before start	~ Minimum	N	~ Maximum cooling
Nest	T_max_nest	44.4 ± 1.25	41.9 ± 1.96	18	− 2.42
	T_mean_nest	37.2 ± 1.16	35.4 ± 1.14	18	− 1.85
	T_min_nest	29.5 ± 3.45	27.9 ± 3.16	18	− 1.60
Cells	T_cell rim_close	37.6 ± 1.49	31.9 ± 2.98	36 (2 cells)	− 5.72
	T_cell center_close	37.7 ± 1.40	33.3 ± 2.49	36 (2 cells)	− 4.38
	T_cell rim_distant	37.3 ± 2.70	36.7 ± 1.81	36 (2 cells)	− 0.67
	T_cell center_distant	38.4 ± 1.75	38.0 ± 1.66	36 (2 cells)	− 0.44
Air	T_a_nest	26.7 ± 5.93	26.9 ± 5.73	18	+ 0.21
Fanner	T_thorax_	35.5 ± 2.58	34.4 ± 2.77	18	− 1.08
	T_head_	35.3 ± 2.30	34.2 ± 2.51	18	− 1.10
	T_abdomen_	33.6 ± 2.80	32.2 ± 3.08	18	− 1.49

Temperatures shown of the whole nest, of specific cells close and distant to the fanners, of the fanners, and of the air close to the nest. Measurements made at 2 points of time each, before start and during ~ minimum temperatures of fanning events. N = 18 or 36 measurements of 9 fanning events (fanners) from 3 nests.

## Discussion

Temperature as a key factor in insect development may delay or even hinder development if it is too low, and may have destructive effects if it is too high^47,48^. The approximately exponential progression of general metabolism and enzymatic function with temperature makes insect brood especially sensitive to environmental changes. High temperatures act mainly on enzymatic structure, which may block development or even lead to death^48^. While solitary insects cannot care much for brood development after egg deposition if environmental conditions change, social insects have more possibilities^14,16^. Vespine wasps and honeybees can use endothermic heat production to stabilize brood temperature, because an insulating nest envelope reduces heat loss^15,17,18^. The open-combed nests of *Polistes *wasps, by contrast, not only loose heat immediately to the environment but also gain it fast^16,20,49^. Our investigation shows how social cooperation nevertheless provides them advanced possibilities of behavioral brood temperature control in comparison to solitary insects.

*Polistes biglumis* has often to cope with low temperatures at night or during bad weather periods (Figs. 4, 6). Since in the breeding season mean temperature is only 11.1 °C in the investigated nesting sites (April–September, 1981–2010^40^) it is especially important for them to compensate for the reduced development speed. Proper nest site choice by the nest foundresses (queens) is the first important behavioral step in spring. Our measurements show that during nighttime the stony substrate or walls where they preferably built their nests on (Fig. 1c) may remain by several degrees warmer than the nest, especially after sunny days. The radiative heat emitted surely heats the nest (Fig. 4a). Concerning the substrate selection for nesting, however, *P. biglumis* seems to be more flexible than shown in our survey. We suggest that it depends on the availability of appropriate stony substrate. While in three locations in the Western Alps only 4 out of 164 nests were built on bushes (*Rosa alpina*)^34^, in Hokkaido in northern Japan *P. biglumis* were observed to nest preferably on *Pinus* trees and shrub plants like *Rubus* and others^10^. In the three Alpine locations in central Europe where we searched for nests in detail in several years, all nests were built on stone.

In the Eastern Alpine habitats we investigated, the ESE nest orientation (mean α = 119°, clockwise from N; Fig. 7) uses the radiative heat of the morning sun for fast nest heating (Figs. 3, 4). This compares well with the orientation of α = 121°, 105° and 138° measured in three locations in the Western Alps^34^. Nest orientation is quite similar to the also open-nesting *P. nimpha*, where mean α = 105° was determined (Ref.^50^; Fig. S7). Using solar heat to speed up brood development allows *P. biglumis* to breed successfully also in the harsh climate in Oketo in northern Hokkaido^10^. The lower mean annual temperatures of ~ 3.5 °C there in comparison to ~ 5.4 °C in our investigated Alpine sites, however, does not mean harsher conditions for brood development. The mean temperature of ~ 11.9 °C in the Oketo breeding season (April–September^51^) is quite similar to the mean of our nesing sites (~ 11.1 °C^40^).

The nearly 10 °C higher mean environmental temperatures *P. gallicus* experiences in the breeding season (~ 20.8 °C; April–September, 1981–2010^41^) in comparison to *P. biglumis* (~ 11.1 °C^40^) reduces the pressure to compensate for reduced development speed. Rather, this quite high value, with extremes of more than 40 °C in midsummer, promotes the avoidance of sunny nesting sites. Therefore, nest orientation can be more variable (Fig. 7) because capture of solar radiation for nest thermoregulation is not desired in most nests. Rather, the danger of overheating is great on hot days and the main challenge. Building a nest to catch the morning sun (Figs. 4d, 5) may be beneficial for fast colony development in early spring when air temperatures are low. To use this advantage, however, nesting in rather close vicinity to consistent water sources is indispensable (our own observations). The behavioral plasticity of *P. gallicus* is reflected in the flexibility concerning nesting site choice: the bimodal distribution we found, with α = 134° (~ SE) and α = 32° (~ NNE) (Fig. 7), differs from the mean orientation of α = 102° in a Spanish population nesting on plants^39^, and α = 150° in Spanish urban environments^52^. The ~ NNE orientation of part of our nests was not the result of the downward orientation of some of them (see Fig. S8).

Using the sun for increasing body and nest temperature is by far more important in the cooler Alpine habitats of *P. biglumis* than in the hot southern habitats of *P. gallicus*. This way, *P. biglumis* is able to increase its body temperature up to 39 °C on warm days (Fig. 6a). A high body temperature improves agility and readiness for flight, which is especially important in the lower range of air temperatures where they are able to fly out (> 18–20 °C). In *P. gallicus*, by contrast, the generally higher temperatures and the more frequent occurrence of hot days makes the adults different troubles of thermoregulation. On hot days their body temperature may reach 45–46 °C even in shade (Fig. 6b). While the body temperature in *P. biglumis* (< 40 °C; Fig. 6a) remains in a safe distance from their critical thermal maximum (CT_max_) of 47.2 °C (our unpublished observation), the body temperature of *P. gallicus* of up to 45–46 °C (Fig. 6b) may increase close to it (CT_max_ = 47.6 °C^25^). On hot days, with T_a_nest > 35 °C, *P. gallicus* adults were observed to cool their head by crawling into cool cells where evaporation of water had led to a temperature drop (Ref.^25^; compare Fig. 6f). We suggest that this helps to save their central neural tissues from thermal damage. To minimize the challenge during such hot days they start collection of water for evaporative cooling already at moderate temperatures (Fig. 5).

A main question addressed in this investigation is: which is the dominant measure of nest temperature control in different climates, fanning or evaporative cooling with water? *P. gallicus* adults were able to keep the temperature of their brood below ~ 42.5 °C (Fig. 6d) despite critically high body temperatures (Fig. 6b). The decrease of brood temperature to up to 10 °C below T_a_nest demonstrates the effectiveness of their cooling measures. In the upper range of ambient temperatures they experience, brood temperature shows striking similarity to that in the upper range of *P. biglumis* high up in the mountains (Fig. 6c). Though both species obviously own the full repertoire of behaviors for nest temperature control, the climatic differences promote a different weighting of their use. *P. gallicus* uses predominantly evaporative cooling with water drops to prevent overheating of the brood. Water droplet temperature of up to 15 °C below T_a_nest (up to ~ 10 °C on average; Fig. 6f) effectively cools the nest (Fig. 6d). Fanning is quite rare, because it would produce no cooling effect to fan the hot ambient air onto a warm nest. Only in case of the sun shining on a nest for a longer time, which may drive the nest temperature to values higher than 50 °C, fanning makes sense (Figs. 4d, 5c).

In the cool climate *P. biglumis* experiences the situation is different. On the one hand, an increase of the brood temperature above ambient levels is of benefit because it speeds up brood development. On the other hand, too high temperatures may soon lead to destructive effects on metabolism^48^. Therefore, *P. biglumis* starts active counter-cooling soon after sunrise (Figs. 3d,e, 4a,b)^20^. Convective cooling by fanning usually starts first. It is quite efficient even during the times of highest nest temperatures, because in the cool Alpine climate the temperature of the ambient air remains always considerably below the temperature of a nest heated by the sun (Figs. 4a,b, 8, Figs. S5, S6). We suggest that the short flights around the nest sometimes observed between fanning events and cell inspections (Video S1), already reported by Steiner^20^, contribute to air mixing around the nest. It is unknown whether they might have a communicative function, e.g. to activate nestmates for nest temperature control. Collection of water is usually started and done by the *P. biglumis* queen but helper wasps may contribute to this task on demand^20^. It is probably especially important in foundress nest where no helpers are present for fanning when the queen leaves the nest. As soon as evaporative cooling is no longer necessary because the nest temperature decreases (e.g. after sunset) water drops are actively removed: the wasps suck them up and let the drops fall from the nest^20^. Despite the differences in climate and the differential weighting of thermoregulatory behaviors between *P. biglumis* and *P. gallicus*, both species rely strongly on the presence of nearby water sources for successful breeding.

A multifactor ANOVA comparison of nest and body temperatures between the two species, compensating for the main identified environmental effects (= covariables: T_a_nest, Radiation, T_substrate_; Table S5) uncovered interesting similarity between *P. biglumis* and *P. gallicus*, despite the ~ 10 °C difference in mean annual and breeding season temperature. The finding that mean nest temperatures (T_nest_(mean)) compensated for the environmental covariables (see ‘Mean T’ in Table S5) did not differ between species (P = 0.09) underpins the wasps’ efforts to establish similar thermal conditions for the brood, despite the differences in climate. The finding that ‘compensated’ temperatures (‘mean T’ in Table S5) of the water drops for nest cooling (T_water_) were not higher but even somewhat lower in *P. gallicus* inhabiting the considerably warmer climate demonstrates the effectiveness of their behavioral measures of brood temperature control.

Climate is a main driver of insect dispersion and development in a warming world^53,54^. Though the high behavioral plasticity of *P. biglumis* and *P. gallicus* allows compensation of a broad variation of short-term environmental effects, the rather strict separation of their current distribution ranges suggests that behavioral plasticity alone may not be sufficient to allow these wasps settle in their current distribution ranges in a warmer future climate scenario. To withstand long-term environmental changes, physiological traits may be equally important. Adults of *Polistes* species from different climates were shown to differ in their level and sensitivity to thermal variation of their respiratory metabolism^25,36,37^. Since the brood lacks the behavioral possibilities of thermoregulation of the adults, investigation of their physiological traits like sensitivity to high temperature are important to allow assessment of these wasps’ dispersion in a warmer future climate scenario.

## Conclusion

In *P. biglumis* from cool Alpine climate and *P. gallicus* from warm Mediterranean climate behavioral plasticity allows flexible control of brood temperature below a maximum of 40–42 °C. The cool environment induces *P. biglumis* to seek the morning sun for nest site choice, and to prevent overheating of the brood by fanning and evaporative cooling with water drops. The warm environment of *P. gallicus* promotes the choice of shaded nesting sites, and induces cooling of the brood with water drops and avoidance of fanning on hot days.

## Supplementary Information

Supplementary Information.

Supplementary Video S1.
