# Supplementary material for: Effect of climate on strategies of nest and body temperature regulation in paper wasps, Polistes biglumis and Polistes gallicus
Source: Sci Rep. 2022 Mar 1;12:3372. doi: 10.1038/s41598-022-07279-0 (PMC8888551; doi:10.1038/s41598-022-07279-0)
Supplement: Supplementary file 1 — Supplementary Information. [file 41598_2022_7279_MOESM1_ESM.pdf]

Supplementary materials

Effect of climate on strategies of nest and body temperature regulation in paper wasps, *Polistes biglumis* and *Polistes gallicus*

Anton Stabentheiner[1], Julia Magdalena Nagy[1,2], Helmut Kovac[1], Helmut Käfer[1], Iacopo Petrocelli[3], Stefano Turillazzi[3]

[1]Institute of Biology, University of Graz, Universitätsplatz 2, 8010 Graz, Austria. [2]Department of Neuroscience and Developmental Biology, University of Vienna, Djerassiplatz 1, 1030 Wien, Austria.[3]Dipartimento di Biologia, Università di Firenze, Via Madonna del Piano 6, 50019 Sesto Fiorentino, Italy. Correspondence and requests for materials should be addressed to A.S. (e-mail: anton.stabentheiner@uni-graz.at) or H.KO. (e-mail: helmut.kovac@uni-graz.at)

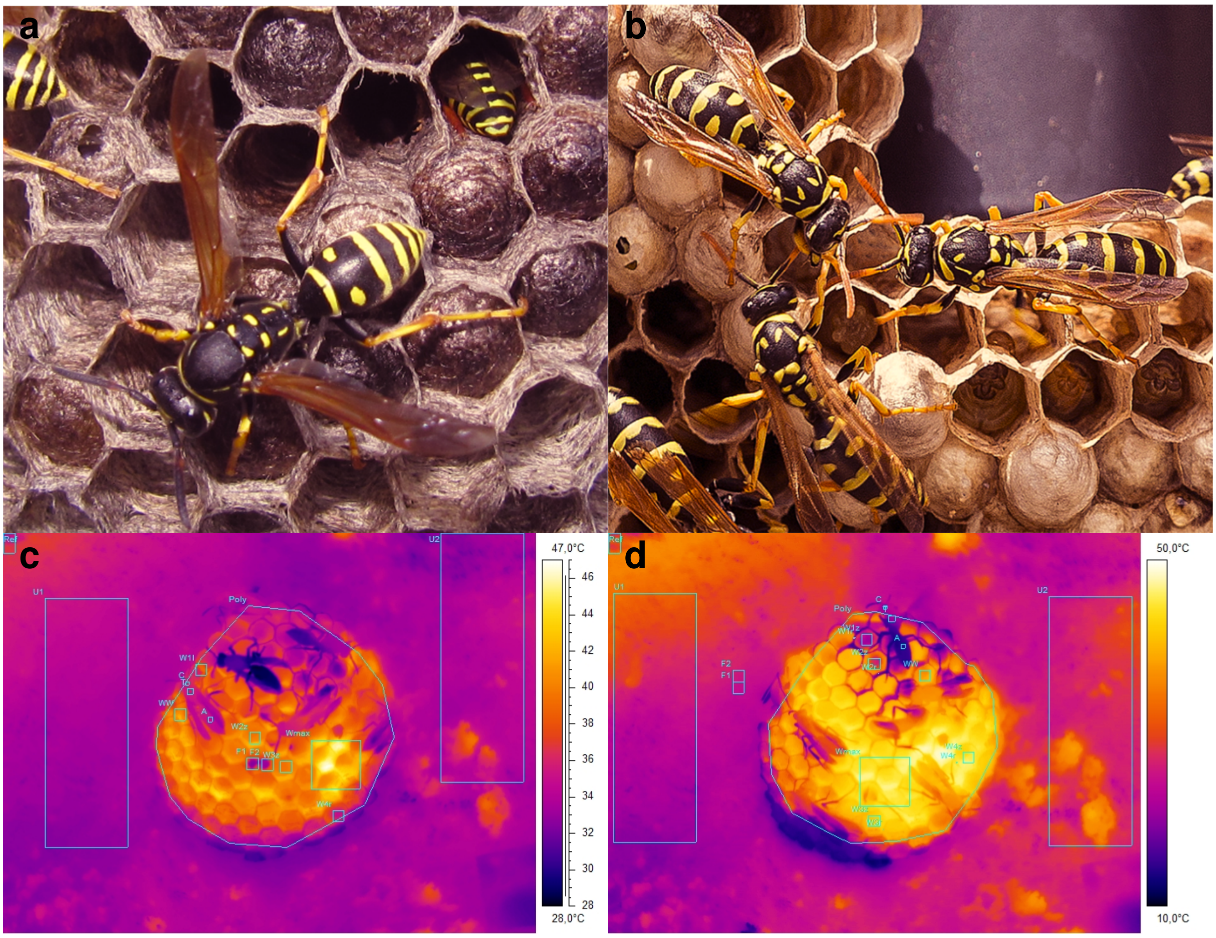

Figure S1. Wasps and definition of evaluation tools. (**a**) *Polistes biglumis*, (**b**) *Polistes gallicus*. Exemplary positioning and size of measurement tools during: (**c**) measurement of body and nest temperatures, (**d**) evaluation of fanning events. Use of measurement tools: (**c, d**) Poly = whole nest (wasps and cells); Wmax = max. nest temperature; U1, U2 = substrate; C = caput (head), T = thorax, A = abdomen; F1, F2 = water droplets (if present); WW = cell near wasp; Ref = reference radiator (if in picture); (**c**) W1l, W2z, W3z, W4r = cell interior (brood) diagonal from left nest edge to center and right edge; (**d**) W1r, W2r, W3r, W4r = cell rims close and distant to the fanner, and W1z, W2z, W3z, W4z = cell interiors close and distant to the fanner.

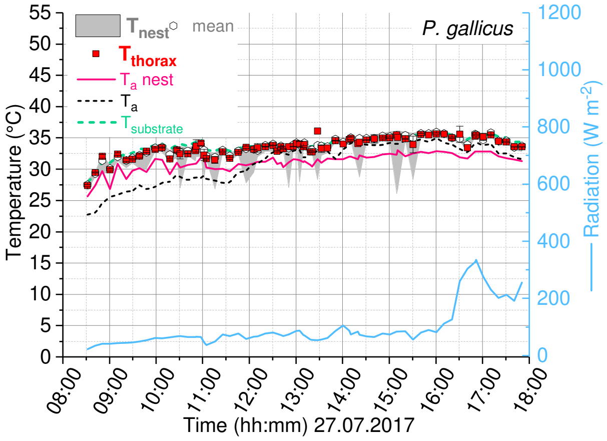

Figure S2. Example of daily temperature changes of a nest and wasps of *P. gallicus*. T_{thorax} = mean thorax surface temperature of up to five adult individuals per time of measurement; gray ribbon: total range of nest temperatures (T_{max} : T_{min}) with mean; $T_{substrate}$ = temperature beside the nest; $T_a$nest = ambient air temperature directly at the nest. T_a = ambient air temperature in shade 1-3m away from the nest; Radiation = global radiation hitting the nest. Fanning was never observed! Time = CEST = UTC+2h.

https://doi.org/10.1038/s41598-022-07279-0

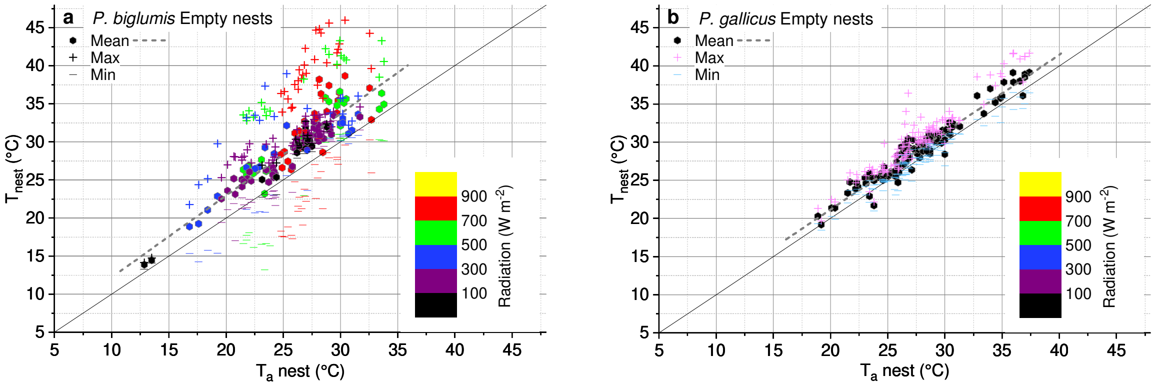

Figure S3. Temperature of empty nests of *P. biglumis* (a) and *P. gallicus* (b) in dependence on ambient temperature close to the nest (T_a nest) and global radiation. Regression lines: **a**) Mean T_{nest} = 1.43936 + 1.07598 * $T_a$nest (corr. R^2 = 0.83528, P<<0.0001); **b**) Mean T_{nest} = 0.89998 + 1.01364 * $T_a$nest (corr. R^2 = 0.93876, P<<0.0001). Thin diagonal lines = isolines.

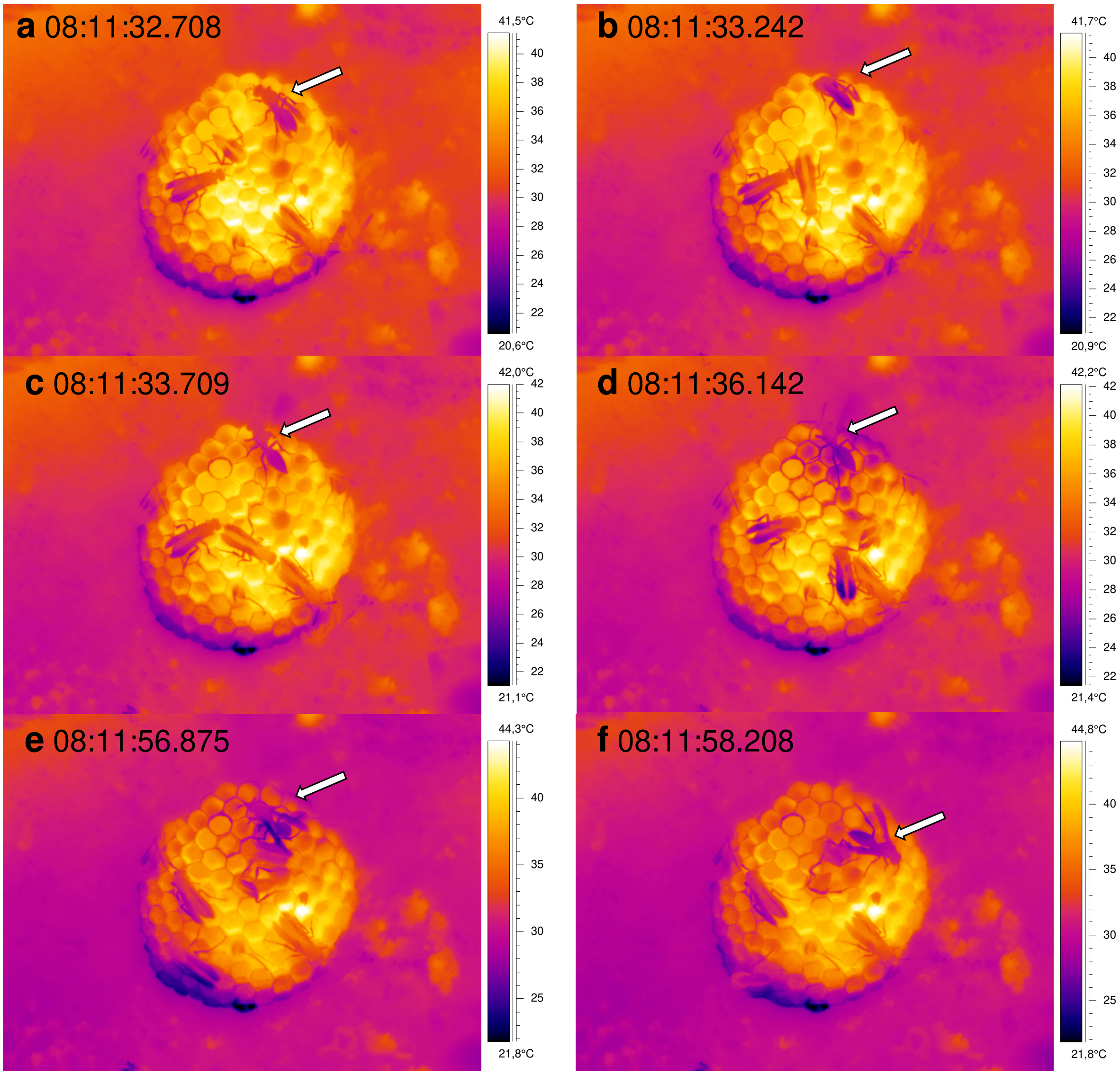

Figure S4. Behavior of *P. biglumis* during nest cooling. Top wasp (see arrows): **a**) sitting on the nest; **b**) inspecting a cell interior; **c**, **d**) fanning, with cooled cells around the fanner; **e**) immediately after stop of fanning; **f**) the fanner walking hectically across the nest. Time = CEST = UTC+2h. See also Video S1 for a thermographic real-time recording of behaviors.

https://doi.org/10.1038/s41598-022-07279-0

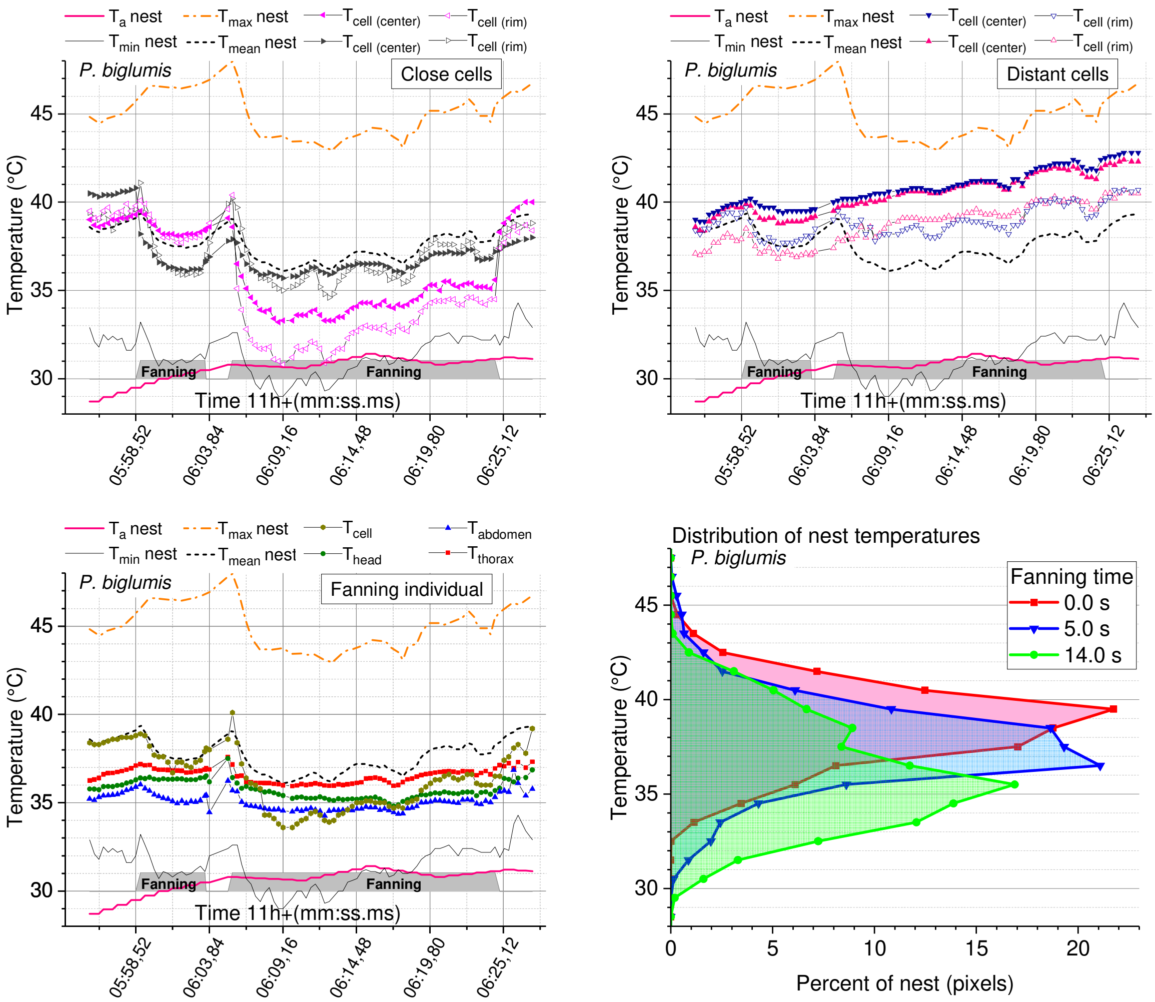

Figure S5. Cooling effect of fanning in a *Polistes biglumis* nest. Temperatures measured on cell rims and centers of cells close to and distant of the fanner, and of the body temperature of the fanner. Note the fast temperature increases in close cells after stops of fanning. Also shown are maximum, mean and minimum nest temperature, air temperature close to the nest ($T_a$nest), and the change of the total distribution of nest temperatures during different times of fanning (percent of nest at a certain temperature estimated from number of pixels in "Poly" in Fig. S2d). Time = CEST = UTC+2h. Gray bars = duration of fanning. For more samples see Fig. 8 and Fig. S6.

https://doi.org/10.1038/s41598-022-07279-0

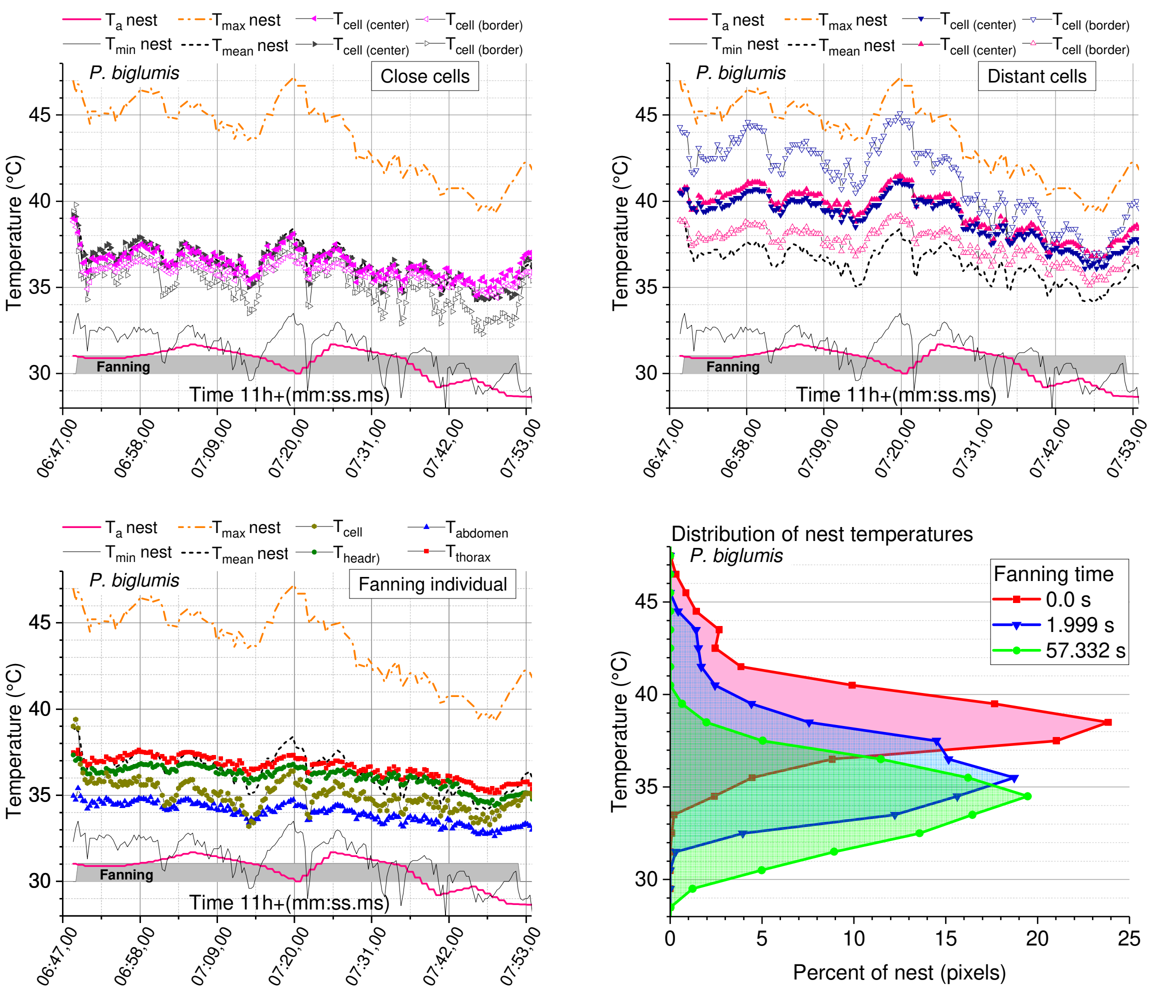

Figure S6. Cooling effect of fanning in a *Polistes biglumis* nest. Temperatures measured on cell rims and centers of cells close to and distant of the fanner, and of the body temperature of the fanner. Also shown are maximum, mean and minimum nest temperature, air temperature close to the nest ($T_a$nest), and the change of the total distribution of nest temperatures during different times of fanning (percent of nest at a certain temperature estimated from number of pixels in "Poly" in Fig. S2d). Gray bars = duration of fanning. Time = CEST = UTC+2h. For more samples see Fig. 8 and Fig. S5.

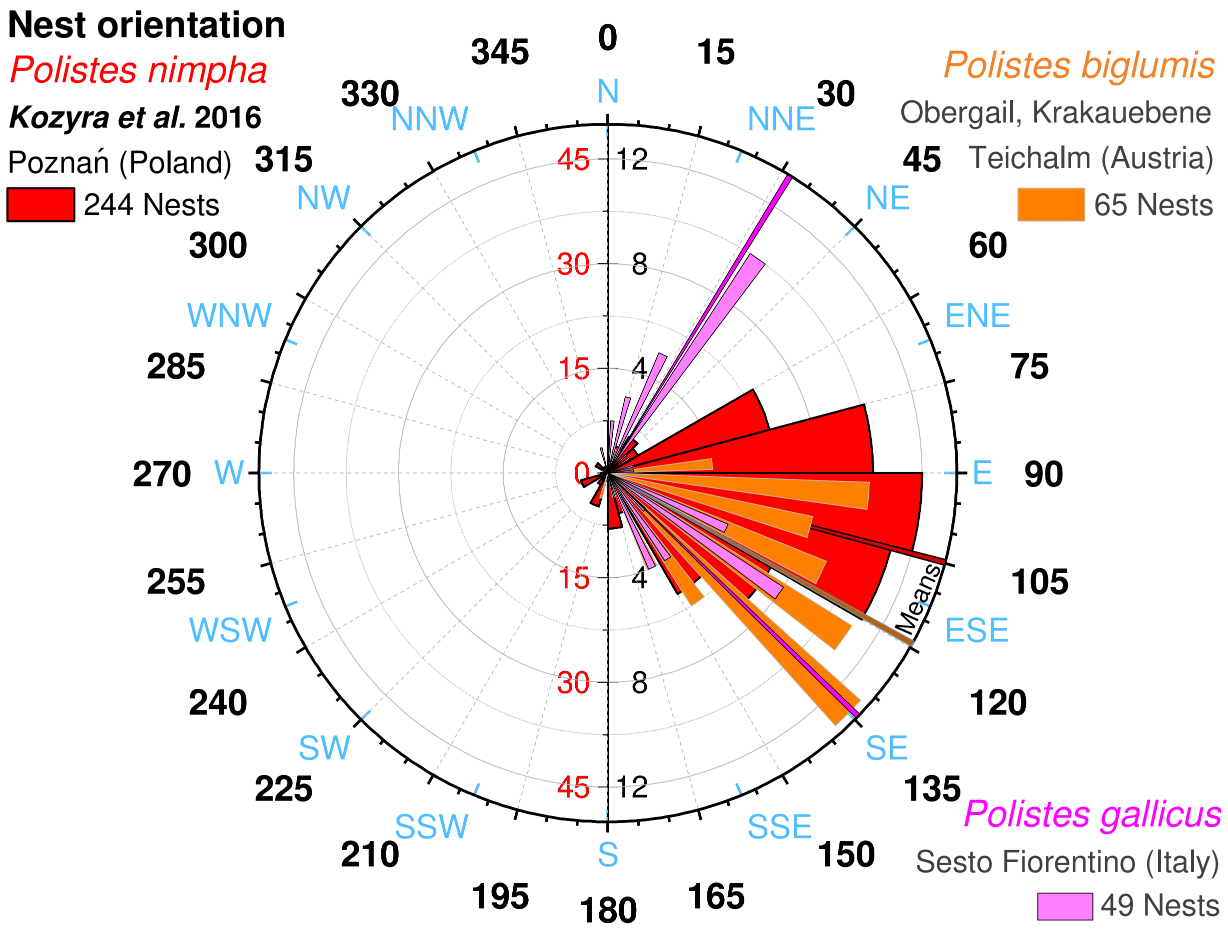

Figure S7. Nest orientation of *Polistes biglumis* in Austria and *Polistes gallicus* in Italy (this paper, see Fig. 7; black scale) in comparison to *Polistes nimpha* in Poland (measured by Kozyra et al. [50]; red scale). Mean of *P. nimpha* estimated from angular class means weighted by numbers in classes in Kozyra et al. [50], according to Batschelet [46]. Thin bars = means.

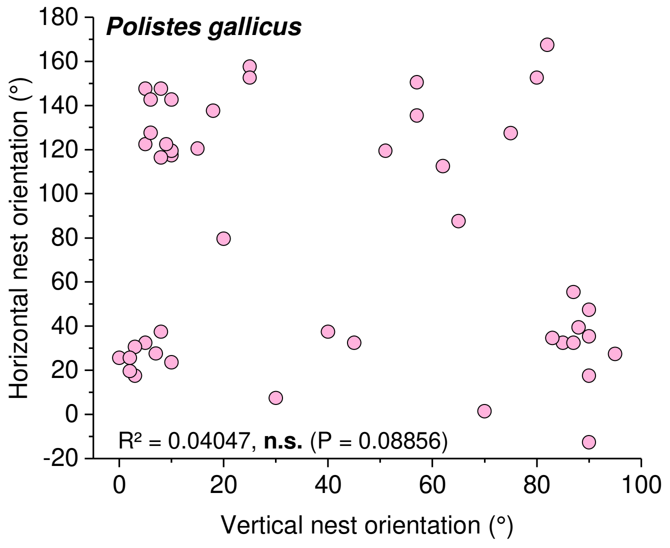

Figure S8. Correlation of horizontal and vertical nest orientetion in *Polistes gallicus*. Horizontal and vertical orientation measured clockwise from N and downward from horizontal, respectively. See also Fig. 7 and Fig. S7.

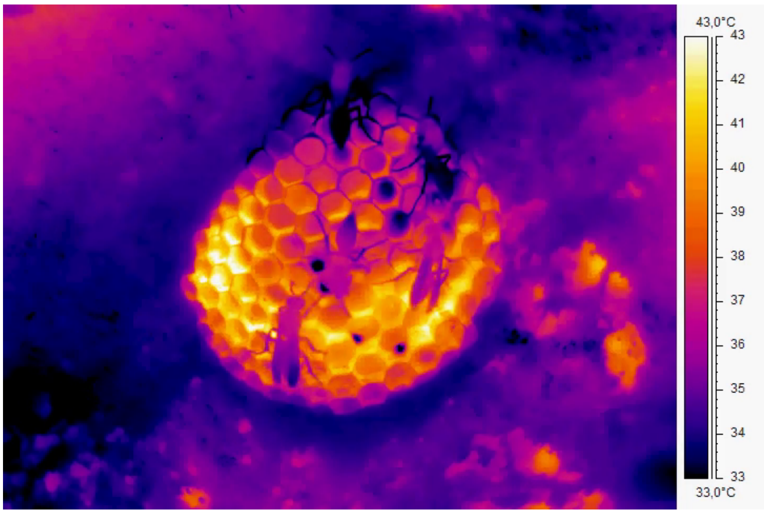

Video S1. Thermographic real-time recording of nest cooling behaviors of *Polistes biglumis* wasps. FLIR22866_X2_32-43-30HZ.m4v

Table S1. Nest statistics for thermographic measurements in *Polistes biglumis* and *Polistes gallicus*. F2 = foundress nest with 1 egg.

Species	Location	Date (d m y)	Nest	N_{cells}	N_{wasps}	Eggs	Larvae	Pupae
P. biglumis	Obergail	20-21 Aug 2010	N1	90	12	✓	✓	✓
		20-21 Aug 2010	N2	45	8	✓	✓	✓
		20-21 Aug 2010	N3	36	8	-	✓	✓
	Obergail	16-18 Jul 2017	W4	95	9	✓	✓	✓
		15-16, 19-20 Jul 2017	W5	99	8	✓	✓	✓
		21 Jul 2017	S4	48	4	✓	✓	✓
	Obergail	28-30 Apr 2018	F2	18	1	✓	-	-
	Mean			61.6	7.1			
	SD			32.50	3.58			
	N			7	7			
P. gallicus	Sesto Fiorentino	4-5 Aug 2015	N1	162	6	✓	✓	-
		4-5 Aug 2015	N2	19	4	✓	✓	-
	Sesto Fiorentino	26 Jul 2017	N1	180	21	✓	✓	✓
		27 Jul 2017	N2	35	8	-	✓	✓
		31 Jul, 1 Aug 2017	N3	381	64	✓	✓	✓
		28 Jul 2017	N4	23	4	✓	✓	✓
	Sesto Fiorentino	12-13 Jun 2018	N1	136	14	✓	✓	✓
		13 Jun 2018	N2	28	2	✓	✓	✓
		14 Jun 2018	N3	30	1	✓	✓	✓
		15 Jun 2018	N4	23	2	-	✓	✓
	Mean			101.7	12.6			
	SD			117.00	19.11			
	N			10	10			

https://doi.org/10.1038/s41598-022-07279-0

Table S2. Regression statistics for Fig. 6. Regressions were calculated for shaded conditions (Radiation = 0-100 W/m^2) and sunshine (Radiation > 100 W/m^2).

Polistes biglumis			***Polistes gallicus***		
**Fig 6a:** 0-100 W/m^2			**Fig 6b:** 0-100 W/m^2		
Function	y = a*(1 + (d-1)*exp(-k*(x-xc)))^(1/(1-d))		Function	y = a*(1 + (d-1)*exp(-k*(x-xc)))^(1/(1-d))	
	**Parameters**	**Value**		**Parameters**	**Value**
**N**	a	33.30038	**N**	a	43.1248
1182	xc	17.30062	1849	xc	25.64835
**R^2** (adj. for df)	d	2.66866	**R^2** (adj. for df)	d	4.04607
0.9524	k	0.19444	0.96239	k	0.16561
**df**	**F value**	**P**	**df**	**F value**	**P**
4	50655.67857	0	4	415224.40331	0
**Fig 6a:** >100 W/m^2			**Fig 6b:** >100 W/m^2		
Function	y = a*(1 + (d-1)*exp(-k*(x-xc)))^(1/(1-d))		Function	y = a*(1 + (d-1)*exp(-k*(x-xc)))^(1/(1-d))	
	**Parameters**	**Value**		**Parameters**	**Value**
**N**	a	39.78362	**N**	a	46.88662
604	xc	-1.72616	459	xc	25.02548
**R^2** (adj. for df)	d	5.51901E-6	**R^2** (adj. for df)	d	3.13224
0.3951	k	0.06693	0.88466	k	0.13799
**df**	**F value**	**P**	**df**	**F value**	**P**
4	18262.72032	0	4	44034.18034	0
**Fig 6c:** 0-100 W/m^2			**Fig 6d:** 0-100 W/m^2		
Function	y = a*(1 + (d-1)*exp(-k*(x-xc)))^(1/(1-d))		Function	y = a/(1 + b*exp(-k*x))	
	**Parameters**	**Value**		**Parameters**	**Value**
**N**	a	31.6623	**N**	a	37.25805
446	xc	19.43795	372	b	26.71312
**R^2** (adj. for df)	d	5.12031	**R^2** (adj. for df)	k	0.17399
0.94268	k	0.34228	0.7713		
**df**	**F value**	**P**	**df**	**F value**	**P**
4	18552.70996	0	3	47060.11861	0
**Fig 6c:** >100 W/m^2			**Fig 6d:** >100 W/m^2		
Function	y = a/(1 + b*exp(-k*x))		Function	y = a/(1 + b*exp(-k*x))	
	**Parameters**	**Value**		**Parameters**	**Value**
**N**	a	37.23623	**N**	a	39.78673
444	b	0.78158	116	b	19.47444
**R^2** (adj. for df)	k	0.07687	**R^2** (adj. for df)	k	0.15554
0.07038			0.46564		
**df**	**F value**	**P**	**df**	**F value**	**P**
3	9864.88997	0	3	16852.80637	0
**Fig 6e:** 0-100 W/m^2			**Fig 6f:** 0-100 W/m^2		
Function	y = a + b*x		Function	y = a*(1 + (d-1)*exp(-k*(x-xc)))^(1/(1-d))	
	**Parameters**	**Value**		**Parameters**	**Value**
**N**	a	-0.65503	**N**	a	37.01784
28	b	1.0877	474	xc	6.88713
**R^2** (adj. for df)			**R^2** (adj. for df)	d	8.0175E-8
0.54571			0.01119	k	0.07827
**df**	**F value**	**P**	**df**	**F value**	**P**
1	33.43339	4.32604E-6	4	161615.12461	0
**Fig 6e:** >100 W/m^2			**Fig 6f:** >100 W/m^2		
Function	y = a/(1 + b*exp(-k*x))		Function	y = a + b*x	
	**Parameters**	**Value**		**Parameters**	**Value**
**N**	a	33.94687	**N**	a	33.00219
565	b	25.43604	1723	b	0.04674
**R^2** (adj. for df)	k	0.2732	**R^2** (adj. for df)		
0.3134			0.4697		
**df**	**F value**	**P**	**df**	**F value**	**P**
3	34170.10356	0	1	5.34144	0.02125

https://doi.org/10.1038/s41598-022-07279-0

Table S3. Multiple linear model regressions of wasp body, cell, brood, water and nest temperatures in relation to air temperature at the nest ($T_a$nest) and radiation. T_{cell}(wasp) = cell temperature close to the measured wasps; T_{pupae} = closed cells (pupae and praepupae).

Polistes biglumis					***Polistes gallicus***				
**Model:**	T_{thorax} = 0.663091 + 1.05429*$T_a$nest + 0.0073271*Radiation				**Model:**	T_{thorax} = 4.41488 + 0.897436*$T_a$nest + 0.0038995*Radiation			
**Parameters:**	Constant	$T_a$nest	Radiation	N	**Parameters:**	Constant	$T_a$nest	Radiation	N
	0.663091	1.05429	0.0073271	1798		4.41488	0.897436	0.0038995	2488
**P:**	0.0171	0	0		**P:**	0	0	0	
**ANOVA:**	**df**	R^2 (%, adj. for df)	**F quotient**	**P**	**ANOVA:**	**df**	R^2 (%, adj. for df)	**F quotient**	**P**
	2	87.9143	6536.9	0		2	93.7527	18662.18	0
**Model:**	T_{head} = 0.801792 + 1.04687*$T_a$nest + 0.00674879*Radiation				**Model:**	T_{head} = 4.98728 + 0.874044*$T_a$nest + 0.00342588*Radiation			
**Parameters:**	Constant	$T_a$nest	Radiation	N	**Parameters:**	Constant	$T_a$nest	Radiation	N
	0.801792	1.04687	0.00674879	1786		4.98728	0.874044	0.00342588	2480
**P:**	0.0036	0	0		**P:**	0	0	0	
**ANOVA:**	**df**	R^2 (%, adj. for df)	**F quotient**	**P**	**ANOVA:**	**df**	R^2 (%, adj. for df)	**F quotient**	**P**
	2	87.6588	6340.36	0		2	93.0003	16469.44	0
**Model:**	$T_{abdomen}$ = 0.0755071 + 1.0775*$T_a$nest + 0.00538768*Radiation				**Model:**	$T_{abdomen}$ = 4.09628 + 0.909778*$T_a$nest + 0.00281136*Radiation			
**Parameters:**	Constant	$T_a$nest	Radiation	N	**Parameters:**	Constant	$T_a$nest	Radiation	N
	0.0755071	1.0775	0.00538768	1796		4.09628	0.909778	0.00281136	2483
**P:**	0.7829	0	0		**P:**	0	0	0	
**ANOVA:**	**df**	R^2 (%, adj. for df)	**F quotient**	**P**	**ANOVA:**	**df**	R^2 (%, adj. for df)	**F quotient**	**P**
	2	87.2386	6136.43	0		2	93.763	18657.46	0
**Model:**	T_{cell}**(wasp)** = 1.56651 + 1.05861*$T_a$nest + 0.00760077*Radiation				**Model:**	T_{cell}**(wasp)** = 9.69614 + 0.70718*$T_a$nest + 0.00464809*Radiation			
**Parameters:**	Constant	$T_a$nest	Radiation	N	**Parameters:**	Constant	$T_a$nest	Radiation	N
	1.56651	1.05861	0.00760077	1798		9.69614	0.70718	0.00464809	2728
**P:**	0	0	0		**P:**	0	0	0	
**ANOVA:**	**df**	R^2 (%, adj. for df)	**F quotient**	**P**	**ANOVA:**	**df**	R^2 (%, adj. for df)	**F quotient**	**P**
	2	83.8733	4673.99	0		2	81.5032	6009.05	0
**Model:**	T_{egg} = 4.73166 + 0.733708*$T_a$nest + 0.0115997*Radiation				**Model:**	T_{egg} = 47.3688 - 0.168815*$T_a$nest - 0.0732512*Radiation			
**Parameters:**	Constant	$T_a$nest	Radiation	N	**Parameters:**	Constant	$T_a$nest	Radiation	N
	4.73166	0.733708	0.0115997	133		47.3688	-0.168815	-0.0732512	16
**P:**	0.0012	0	0		**P:**	0.0008	0.5244	0.4459	
**ANOVA:**	**df**	R^2 (%, adj. for df)	**F quotient**	**P**	**ANOVA:**	**df**	R^2 (%, adj. for df)	**F quotient**	**P**
	2	86.0359	407.64	0		2	0	0.5	***0.6164***
**Model:**	T_{larvae} = 4.28143 + 0.942742*$T_a$nest + 0.00664672*Radiation				**Model:**	T_{larvae} = 17.8605 + 0.474338*$T_a$nest + 0.00487993*Radiation			
**Parameters:**	Constant	$T_a$nest	Radiation	N	**Parameters:**	Constant	$T_a$nest	Radiation	N
	4.28143	0.942742	0.00664672	400		17.8605	0.474338	0.00487993	274
**P:**	0	0	0		**P:**	0	0	0	
**ANOVA:**	**df**	R^2 (%, adj. for df)	**F quotient**	**P**	**ANOVA:**	**df**	R^2 (%, adj. for df)	**F quotient**	**P**
	2	77.8072	700.44	0		2	62.8895	232.32	0
**Model:**	T_{pupae}= 3.44504 + 0.940025*$T_a$nest + 0.00528399*Radiation				**Model:**	T_{pupae} = 17.8605 + 0.713172*$T_a$nest + 0.00556407*Radiation			
**Parameters:**	Constant	$T_a$nest	Radiation	N	**Parameters:**	Constant	$T_a$nest	Radiation	N
	3.44504	0.940025	0.00528399	397		9.47043	0.713172	0.00556407	973
**P:**	0.0003	0	0		**P:**	0	0	0	
**ANOVA:**	**df**	R^2 (%, adj. for df)	**F quotient**	**P**	**ANOVA:**	**df**	R^2 (%, adj. for df)	**F quotient**	**P**
	2	74.4985	579.42	0		2	78.5835	1784.28	0
**Model:**	T_{water} = 24.0442 + 0.214143*$T_a$nest + 0.0038388*Radiation				**Model:**	T_{water} = 22.2658 + 0.293081*$T_a$nest + 0.00251734*Radiation			
**Parameters:**	Constant	$T_a$nest	Radiation	N	**Parameters:**	Constant	$T_a$nest	Radiation	N
	24.0442	0.214143	0.0038388	612		22.2658	0.293081	0.00251734	552
**P:**	0	0	0		**P:**	0	0	0	
**ANOVA:**	**df**	R^2 (%, adj. for df)	**F quotient**	**P**	**ANOVA:**	**df**	R^2 (%, adj. for df)	**F quotient**	**P**
	2	28.1329	120.59	0		2	40.8158	191	0
**Model:**	T_{nest}**(max)** = 5.6148 + 0.920164*$T_a$nest + 0.0148126*Radiation				**Model:**	T_{nest}**(max)** = 2.73006 + 0.976603*$T_a$nest + 0.0101429*Radiation			
**Parameters:**	Constant	$T_a$nest	Radiation	N	**Parameters:**	Constant	$T_a$nest	Radiation	N
	5.6148	0.920164	0.0148126	1888		2.73006	0.976603	0.0101429	2736
**P:**	0	0	0		**P:**	0	0	0	
**ANOVA:**	**df**	R^2 (%, adj. for df)	**F qoutient**	**P**	**ANOVA:**	**df**	R^2 (%, adj. for df)	**F qoutient**	**P**
	2	82.5138	4453.18	0		2	91.5042	14729.72	0
**Model:**	T_{nest}**(mean)** = 2.38935 + 1.0085*$T_a$nest + 0.00696722*Radiation				**Model:**	T_{nest}**(mean)** = 6.58421 + 0.821208*$T_a$nest + 0.00265919*Radiation			
**Parameters:**	Constant	$T_a$nest	Radiation	N	**Parameters:**	Constant	$T_a$nest	Radiation	N
	2.38935	1.0085	0.00696722	2524		6.58421	0.821208	0.00265919	2980
**P:**	0	0	0		**P:**	0	0	0	
**ANOVA:**	**df**	R^2 (%, adj. for df)	**F quotient**	**P**	**ANOVA:**	**df**	R^2 (%, adj. for df)	**F quotient**	**P**
	2	82.3024	5867.6	0		2	83.5367	7558.88	0

https://doi.org/10.1038/s41598-022-07279-0

Table S4. Multiple linear model regressions of wasp body, cell, brood, water and nest temperatures in relation to air temperature at the nest ($T_a$nest), radiation and substrate temperature ($T_{substrate}$). T_{cell}(wasp) = cell temperature close to the measured wasps; T_{pupae} = closed cells (pupae and praepupae).

Polistes biglumis

**Model:**	**T_{thorax}** = -2.23641 + 0.69335* $T_a$nest + 0.00616842*Radiation + 0.420965*$T_{substrate}$				
**Parameters:**	Constant	$T_a$nest	Radiation	$T_{substrate}$	N
	-2.23641	0.69335	0.00616842	0.420965	1798
**P:**	0	0	0	0	
**ANOVA:**	**df**	**R^2** (%, adj. for df)	**F qoutient**	**P**	
	3	89.1011	4897.99	0	
**Model:**	**T_{head}** = -2.1428 + 0.678948*$T_a$nest + 0.0055964*Radiation + 0.428729*$T_{substrate}$				
**Parameters:**	Constant	$T_a$nest	Radiation	$T_{substrate}$	N
	-2.1428	0.678948	0.0055964	0.428729	1786
**P:**	0	0	0	0	
**ANOVA:**	**df**	**R^2** (%, adj. for df)	**F qoutient**	**P**	
	3	88.9342	4782.94	0	
**Model:**	**$T_{abdomen}$** = -2.96496 + 0.699074*$T_a$nest + 0.00417238*Radiation + 0.441451*$T_{substrate}$				
**Parameters:**	Constant	$T_a$nest	Radiation	$T_{substrate}$	N
	-2.96496	0.699074	0.00417238	0.441451	1796
**P:**	0	0	0	0	
**ANOVA:**	**df**	**R^2** (%, adj. for df)	**F qoutient**	**P**	
	3	88.6591	4678.56	0	
**Model:**	**T_{cell}(wasp)** = -1.69964 + 0.652027*$T_a$nest + 0.00629557*Radiation + 0.474197*$T_{substrate}$				
**Parameters:**	Constant	$T_a$nest	Radiation	$T_{substrate}$	N
	-1.69964	0.652027	0.00629557	0.474197	1798
**P:**	0	0	0	0	
**ANOVA:**	**df**	**R^2** (%, adj. for df)	**F qoutient**	**P**	
	3	85.275	3469.92	0	
**Model:**	**T_{egg}** = 2.74507 + 0.324319*$T_a$nest + 0.00697478*Radiation + 0.513289*$T_{substrate}$				
**Parameters:**	Constant	$T_a$nest	Radiation	$T_{substrate}$	N
	2.74507	0.324319	0.00697478	0.513289	133
**P:**	0.0505	0.0064	0	0	
**ANOVA:**	**df**	**R^2** (%, adj. for df)	**F qoutient**	**P**	
	3	87.9941	323.49	0	
**Model:**	**T_{larvae}** = 0.296351 + 0.601333*$T_a$nest + 0.00455124*Radiation + 0.464692*$T_{substrate}$				
**Parameters:**	Constant	$T_a$nest	Radiation	$T_{substrate}$	N
	0.296351	0.601333	0.00455124	0.464692	400
**P:**	0.7754	0	0	0	
**ANOVA:**	**df**	**R^2** (%, adj. for df)	**F qoutient**	**P**	
	3	80.0755	535.52	0	
**Model:**	**T_{pupae}** = -0.975249 + 0.545477**$T_a$nest + 0.00302162*Radiation + 0.527851*$T_{substrate}$				
**Parameters:**	Constant	$T_a$nest	Radiation	$T_{substrate}$	N
	-0.975249	0.545477	0.00302162	0.527851	397
**P:**	0.3545	0	0.0001	0	
**ANOVA:**	**df**	**R^2** (%, adj. for df)	**F qoutient**	**P**	
	3	77.6794	460.38	0	
**Model:**	**T_{water}** = 20.282 + 0.184558*$T_a$nest + 0.00239761*Radiation + 0.174133*$T_{substrate}$				
**Parameters:**	Constant	$T_a$nest	Radiation	$T_{substrate}$	N
	20.282	0.184558	0.00239761	0.174133	612
**P:**	0	0	0.0001	0.0002	
**ANOVA:**	**df**	**R^2** (%, adj. for df)	**F qoutient**	**P**	
	3	29.608	86.67	0	
**Model:**	**T_{nest}(max)** = 2.19849 + 0.468458*$T_a$nest + 0.0134079*Radiation + 0.520648*$T_{substrate}$				
**Parameters:**	Constant	$T_a$nest	Radiation	$T_{substrate}$	N
	2.19849	0.468458	0.0134079	0.520648	1888
**P:**	0	0	0	0	
**ANOVA:**	**df**	**R^2** (%, adj. for df)	**F qoutient**	**P**	
	3	83.8313	3262.24	0	
**Model:**	**T_{nest}(mean)** = -2.14936 + 0.510637*$T_a$nest + 0.00475538*Radiation + 0.611022*$T_{substrate}$				
**Parameters:**	Constant	$T_a$nest	Radiation	$T_{substrate}$	N
	-2.14936	0.510637	0.00475538	0.611022	2524
**P:**	0	0	0	0	
**ANOVA:**	**df**	**R^2** (%, adj. for df)	**F qoutient**	**P**	
	3	85.1029	4805.38	0	

Polistes gallicus

**Model:**	**T_{thorax}** = 5.00368 + 0.559154*$T_a$nest + 0.00420989*Radiation + 0.291242*$T_{substrate}$				
**Parameters:**	Constant	$T_a$nest	Radiation	$T_{substrate}$	N
	5.00368	0.559154	0.00420989	0.291242	2488
**P:**	0	0	0	0	
**ANOVA:**	**df**	**R^2** (%, adj. for df)	**F qoutient**	**P**	
	3	95.9768	19777.61	0	
**Model:**	**T_{head}** = 5.59942 + 0.521981*$T_a$nest + 0.00375059*Radiation + 0.303086*$T_{substrate}$				
**Parameters:**	Constant	$T_a$nest	Radiation	$T_{substrate}$	N
	5.59942	0.521981	0.00375059	0.303086	2480
**P:**	0	0	0	0	
**ANOVA:**	**df**	**R^2** (%, adj. for df)	**F qoutient**	**P**	
	3	95.5469	17730.9	0	
**Model:**	**$T_{abdomen}$** = 4.7184 + 0.549191*$T_a$nest + 0.00314149*Radiation + 0.310577*$T_{substrate}$				
**Parameters:**	Constant	$T_a$nest	Radiation	$T_{substrate}$	N
	Apr.84	0.549191	0.00314149	0.310577	2483
**P:**	0	0	0	0	
**ANOVA:**	**df**	**R^2** (%, adj. for df)	**F qoutient**	**P**	
	3	96.2918	21484.48	0	
**Model:**	**T_{cell}(wasp)** = 10.7287 + 0.272718*$T_a$nest + 0.00517888*Radiation + 0.367007*$T_{substrate}$				
**Parameters:**	Constant	$T_a$nest	Radiation	$T_{substrate}$	N
	Okt.87	0.272718	0.00517888	0.367007	2727
**P:**	0	0	0	0	
**ANOVA:**	**df**	**R^2** (%, adj. for df)	**F qoutient**	**P**	
	3	86.096	5627.64	0	
**Model:**	**T_{egg}** = 36.8406 - 0.975044*$T_a$nest + 0.357492*Radiation + 0.423053*$T_{substrate}$				
**Parameters:**	Constant	$T_a$nest	Radiation	$T_{substrate}$	N
	36.8406	-0.975044	0.357492	0.423053	8
**P:**	0.0488	0.0558	0.0679	0.137	
**ANOVA:**	**df**	**R^2** (%, adj. for df)	**F qoutient**	**P**	
	3	51.1285	Mär.44	0.1318	
**Model:**	**T_{larvae}** = 17.861 + 0.100866*$T_a$nest + 0.00428128*Radiation + 0.336978*$T_{substrate}$				
**Parameters:**	Constant	$T_a$nest	Radiation	$T_{substrate}$	N
	17.861	0.100866	0.00428128	0.336978	273
**P:**	0	0.0111	0	0	
**ANOVA:**	**df**	**R^2** (%, adj. for df)	**F qoutient**	**P**	
	3	74.4294	264.91	0	
**Model:**	**T_{pupae}** = 10.0351 + 0.087647*$T_a$nest + 0.00694214*Radiation + 0.552736*$T_{substrate}$				
**Parameters:**	Constant	$T_a$nest	Radiation	$T_{substrate}$	N
	10.0351	0.087647	0.00694214	0.552736	973
**P:**	0	0.0069	0	0	
**ANOVA:**	**df**	**R^2** (%, adj. for df)	**F qoutient**	**P**	
	3	85.1314	1856.09	0	
**Model:**	**T_{water}** = 18.8619 - 0.0278721*$T_a$nest + 0.00165691*Radiation + 0.38456*$T_{substrate}$				
**Parameters:**	Constant	$T_a$nest	Radiation	$T_{substrate}$	N
	18.8619	-0.0278721	0.00165691	0.38456	552
**P:**	0	0.4304	0	0	
**ANOVA:**	**df**	**R^2** (%, adj. for df)	**F qoutient**	**P**	
	3	49.9812	184.53	0	
**Model:**	**T_{nest}(max)** = 3.83154 + 0.485144*$T_a$nest + 0.0107037*Radiation + 0.41755*$T_{substrate}$				
**Parameters:**	Constant	$T_a$nest	Radiation	$T_{substrate}$	N
	3.83154	0.485144	0.0107037	0.41755	2727
**P:**	0	0	0	0	
**ANOVA:**	**df**	**R^2** (%, adj. for df)	**F qoutient**	**P**	
	3	94.7725	16474.91	0	
**Model:**	**T_{nest}(mean)** = 8.01929 + 0.0986386*$T_a$nest + 0.00318776*Radiation + 0.620503*$T_{substrate}$				
**Parameters:**	Constant	$T_a$nest	Radiation	$T_{substrate}$	N
	8.01929	0.0986386	0.00318776	0.620503	2971
**P:**	0	0	0	0	
**ANOVA:**	**df**	**R^2** (%, adj. for df)	**F qoutient**	**P**	
	3	93.6939	14710.16	0	

https://doi.org/10.1038/s41598-022-07279-0

Table S5. Multifactor ANOVA comparison between species. Mean T = mean temperatures after compensation for the effects of covariables. T_{cell}(wasp) = cell temperature close to the measured wasps; T_{pupae} = closed cells (pupae and praepupae).

		N	F-Quotient	P		*P. biglumis*	*P. gallicus*
Dependent variable: $\mathbf{T_{thorax}}$	Factors:						
	Species	4286	4.06	0.0439	**Mean T**:	28.7884	29.0406
	Covariables:				N:	1798	2488
	$T_a$nest		1998.52	0			
	Radiation		2082.41	0			
	$T_{substrate}$		490.05	0			
Dependent variable: $\mathbf{T_{head}}$	Factors:	N	F-Quotient	P		*P. biglumis*	*P. gallicus*
	Species	4266	7.72	0.0055	**Mean T**:	28.5366	28.8848
	Covariables:				N:	1786	2480
	$T_a$nest		1848.18	0			
	Radiation		1807.63	0			
	$T_{substrate}$		506.51	0			
Dependent variable: $\mathbf{T_{abdomen}}$	Factors:	N	F-Quotient	P		*P. biglumis*	*P. gallicus*
	Species	4279	14.67	0.0001	**Mean T**:	28.396	28.8635
	Covariables:				N:	1796	2483
	$T_a$nest		2088.27	0			
	Radiation		1305.22	0			
	$T_{substrate}$		574.64	0			
Dependent variable: $\mathbf{T_{cell}}$**(wasp)**		N	F-Quotient	P		*P. biglumis*	*P. gallicus*
	Species	4526	13.8	0.0002	**Mean T**:	28.5837	29.1748
	Covariables:				N:	1798	2728
	$T_a$nest		702.46	0			
	Radiation		1842.3	0			
	$T_{substrate}$		329.07	0			
Dependent variable: $\mathbf{T_{empty\ cells}}$	Factors:	N	F-Quotient	P		*P. biglumis*	*P. gallicus*
	Species	590	0.81	***0.3676***	**Mean T**:	**30.6195**	**30.1395**
	Covariables:				N:	346	244
	$T_a$nest		49.57	0			
	Radiation		175.66	0			
	$T_{substrate}$		31.29	0			
Dependent variable: $\mathbf{T_{eggs}}$	Factors:	N	F-Quotient	P		*P. biglumis*	*P. gallicus*
	Species	149	0.02	***0.8922***	**Mean T**:	**25.0141**	**24.7202**
	Covariables:				N:	133	16
	$T_a$nest		7.97	0.0054			
	Radiation		33.52	0			
	$T_{substrate}$		21.2	0			
Dependent variable: $\mathbf{T_{larvae}}$	Factors:	N	F-Quotient	P		*P. biglumis*	*P. gallicus*
	Species	674	0.69	***0.4046***	**Mean T**:	**30.7568**	**30.3033**
	Covariables:				N:	400	274
	$T_a$nest		97.92	0			
	Radiation		213.01	0			
	$T_{substrate}$		44.92	0			
Dependent variable: $\mathbf{T_{pupae}}$	Factors:	N	F-Quotient	P		*P. biglumis*	*P. gallicus*
	Species	1370	55.76	0	**Mean T**:	28.2522	30.1069
	Covariables:				N:	397	973
	$T_a$nest		82.33	0			
	Radiation		380.27	0			
	$T_{substrate}$		224.98	0			
Dependent variable: $\mathbf{T_{water}}$	Factors:	N	F-Quotient	P		*P. biglumis*	*P. gallicus*
	Species	1164	6.78	0.0092	**Mean T**:	33.1658	32.3025
	Covariables:				N:	612	552
	$T_a$nest		33.4	0			
	Radiation		55.87	0			
	$T_{substrate}$		61.74	0			
Dependent variable: $\mathbf{T_{nest}}$**(max)**	Factors:	N	F-Quotient	P		*P. biglumis*	*P. gallicus*
	Species	4624	54.12	0	**Mean T**:	32.0823	30.9016
	Covariables:				N:	1888	2736
	$T_a$nest		666.97	0			
	Radiation		4304.59	0			
	$T_{substrate}$		710.92	0			
Dependent variable: $\mathbf{T_{nest}}$**(mean)**	Factors:	N	F-Quotient	P		*P. biglumis*	*P. gallicus*
	Species	5504	2.89	***0.0892***	**Mean T**:	**29.1406**	**29.374**
	Covariables:				N:	2524	2980
	$T_a$nest		484.12	0			
	Radiation		1703.15	0			
	$T_{substrate}$		1409.3	0			

https://doi.org/10.1038/s41598-022-07279-0
